# Metabolic sensing in 3D models of pancreatic ductal adenocarcinoma: probing pH, oxygen, and lactate dynamics to decode chemoresistance

**DOI:** 10.3389/fbioe.2026.1926254

**Published:** 2026-10-12

**Authors:** Anna Chiara Siciliano, Stefania Forciniti, Helena Iuele, Federica Carnevali, Giuseppe Gigli, Loretta L. del Mercato

**Affiliations:** 1 Institute of Nanotechnology, National Research Council (CNR-NANOTEC), Lecce, Italy; 2 Tecnomed Puglia – Technopole for Precision Medicine (Biotech Lecce Hub), Lecce, Italy; 3 Department of Experimental Medicine, University of Salento, Lecce, Italy

**Keywords:** 3D tumor models, chemoresistance, metabolic biosensors, organ-on-chip, pancreatic ductal adenocarcinoma, patient-derived organoids, real-time metabolic monitoring, tumor microenvironment

## Abstract

Pancreatic ductal adenocarcinoma (PDAC) is one of the deadliest malignancies due to its late diagnosis, extensive desmoplasia, and strong chemoresistance. Recent advances in three-dimensional (3D) *in vitro* modelling have provided new insights into tumor-stroma interactions and metabolic adaptations that drive disease progression. This review summarizes the pathogenesis and tumor microenvironment (TME) hallmarks of PDAC, emphasizing the metabolic heterogeneity characterized by altered pH, oxygen, and lactate gradients. It discusses conventional and emerging therapeutic strategies, the molecular basis of drug resistance, and the role of the TME in treatment failure. Furthermore, it highlights the evolution of 3D experimental models as physiologically relevant tools to study PDAC biology and evaluate drug efficacy. Special attention is given to the integration of optical biosensors for non-invasive spatio-temporal monitoring of pH and metabolic fluctuations. These sensing technologies, coupled with advanced bioengineered models, are paving the way for precision oncology and patient-tailored strategies in pancreatic cancer.

## 1 Introduction

PDAC is a deadly and aggressive disease that accounts for more than 90% of all pancreatic cancer cases. It currently ranks as the seventh leading cause of cancer-related deaths worldwide and is projected to become the second within the next decade (Guo et al., 2023). Despite slight advancements in therapeutic strategies, the 5-year survival rate for PDAC remains below 10%, highlighting the urgent need for innovative approaches to advance our understanding of disease biology, diagnosis, and treatment (Pan et al., 2025). PDAC’s dismal prognosis stems from multiple factors, including its asymptomatic early stages, late diagnosis, intrinsic and acquired therapy resistance, and a dense, immunosuppressive, and heterogeneous TME (Luo et al., 2024). A hallmark of PDAC is its highly desmoplastic stroma, which may constitute up to 90% of the tumor mass and is composed of cancer-associated fibroblasts (CAFs), pancreatic stellate cells (PSCs), immune and endothelial cells, and an extracellular matrix (ECM) enriched in collagen, hyaluronic acid, and fibronectin (Guo et al., 2023). This intricate network not only provides structural support but also drives tumor progression, immune evasion, metastasis, and treatment resistance. The PDAC TME is also characterized by pronounced metabolic reprogramming, leading to alterations in pH, oxygen, and metabolite gradients. Poor vascularization, excessive glycolytic activity (the Warburg effect), and inefficient clearance of acidic byproducts such as lactate and protons result in extracellular acidification and chronic hypoxia (Salvador-Barbero et al., 2020). These gradients profoundly influence ECM remodelling, stromal and immune cell behavior, and drug distribution (Mascharak et al., 2023). Hypoxic and acidic conditions promote immune suppression, angiogenesis, and tumor aggressiveness, while lactate accumulation further exacerbates immune evasion, and supports oxidative metabolism in stromal cells (Guo and Wang, 2024). These alterations are mechanistically interconnected, as discussed in detail in Section 3. Hence, real-time sensing of key metabolic parameters such as pH, oxygen, and lactate is essential to understand PDAC biology and therapeutic responses. Optical and electrochemical sensing platforms can provide spatiotemporal insights into these metabolic fluctuations, enabling the characterization of tumor metabolism under dynamic conditions and in response to treatment (Grasso et al., 2023). Conventional two-dimensional (2D) models fail to reproduce the structural and biochemical complexity of the PDAC TME. As a result, there is growing interest in 3D *in vitro* models that better recapitulate *in vivo* architecture and microenvironmental cues (Narayanan et al., 2021). This transition is also supported by recent regulatory developments. In particular, the FDA Modernization Act 2.0, enacted in 2022, encourages the adoption of human-relevant alternative models, including advanced *in vitro* systems, organoids, and organ-on-chip platforms, as complementary approaches to traditional animal studies in preclinical drug development. This evolving regulatory landscape further highlights the increasing importance of physiologically relevant 3D models for translational cancer research (Han, 2023; Zushin et al., 2023). These systems range from multicellular spheroids to advanced bioengineered constructs incorporating multiple cell types and ECM components (Cha et al., 2025; Longati et al., 2013). However, capturing the spatiotemporal metabolic complexity of PDAC, in particular the steep gradients of pH, oxygen, and lactate that drive chemoresistance, requires more than structural fidelity alone. Integrating biosensing technologies into these 3D platforms offers a powerful strategy to monitor metabolic fluctuations in real time and in a non-invasive manner, bridging the gap between structural biomimicry and functional metabolic readouts. Among the available sensing modalities, ratiometric optical sensors based on fluorescent micro- and nanoparticles are particularly attractive, as they enable quantitative, spatially resolved measurements within living 3D constructs without perturbing the system (Grasso et al., 2023; Onesto et al., 2026; Carnevali et al., 2024; Prasad et al., 2021; Fluorescent, 2020). A particularly promising approach uses ratiometric optical pH sensors embedded in alginate-based PDAC scaffolds, allowing non-invasive, real-time monitoring of extracellular acidification following drug exposure (Siciliano et al., 2024; Rizzo et al., 2022). Extending this strategy to include oxygen and lactate sensors would enable a more comprehensive mapping of the metabolic landscape, revealing how hypoxia, acidosis, and lactate accumulation interact to drive tumor progression and drug resistance. Moreover, heterocellular spheroids and organoid-based systems have emerged as powerful tools for studying the immune landscape of PDAC. For example, 3D spheroids comprising cancer cells, PSCs, endothelial cells, and monocytes can generate hypoxic cores and M2-like macrophage phenotypes, faithfully reproducing immune suppression observed *in vivo* (Giustarini et al., 2023). Patient-derived organoids (PDOs) and explants (PDEs) further enable personalized investigations by preserving genetic, phenotypic, and architectural tumor features (Godier et al., 2024). Although previous reviews have separately addressed PDAC metabolism (Ghiglione et al., 2025; Wu et al., 2024), tumor microenvironment biology (Ho et al., 2020; Sherman and Beatty, 2023), advanced 3D culture systems (Jamshidi et al., 2024; Tomás-Bort et al., 2020; Salu and Reindl, 2024), or biosensing technologies (Sharma and Srivastava, 2025; Abuhassan et al., 2026), an integrated perspective specifically focused on the simultaneous sensing of pH, oxygen, and lactate within physiologically relevant 3D PDAC models is still lacking. Here, we specifically focus on the integration of advanced biosensing technologies with engineered 3D PDAC platforms to dynamically monitor extracellular pH, oxygen, and lactate, highlighting their value for investigating tumor–stroma interactions, chemoresistance, therapeutic response, and precision medicine. In this review, we critically discuss recent advances in *in vitro* 3D PDAC models integrated with metabolic sensing technologies, and we compare the currently available *in vitro* platforms, highlighting their complementary advantages, limitations, and translational potential for drug screening and precision oncology.

## 2 PDAC pathogenesis and characteristics

The poor prognosis of PDAC is largely associated with late diagnosis, as nearly 90% of patients present locally advanced disease, and over 50% show systemic metastases (Siegel et al., 2022). Nevertheless, growing evidence indicates that PDAC develops through a multistep process involving potentially detectable non-invasive precursor lesions (Basturk et al., 2015). These include pancreatic intraepithelial neoplasia (PanIN), intraductal papillary mucinous neoplasms (IPMNs), and mucinous cystic neoplasms (MCNs), which differ in their morphology, size, and involvement of the pancreatic ductal system (Felsenstein et al., 2018). PanINs represent the most frequent precursors and are generally microscopic, whereas IPMNs are larger cystic lesions that are often incidentally detected by abdominal imaging and may therefore be subjected to clinical surveillance (Ohtsuka et al., 2024). MCNs are less common and typically occur in the pancreatic body or tail, predominantly in women (Felsenstein et al., 2018). Based on cytological and architectural atypia, precancerous lesions are classified as low-grade or high-grade dysplasia (Basturk et al., 2015). In IPMNs, dysplasia grade correlates with histologic subtype: intestinal and pancreatobiliary types are usually high-grade, whereas gastric-type IPMNs tend to be low-grade (Oyama et al., 2020). High-grade precursors are significantly associated with PDAC development, emphasizing their role as direct precursors of invasive carcinoma (Rezaee et al., 2016). Despite well-established histopathological classifications, the cell of origin in PDAC remains debated. PanIN development is commonly linked to acinar-to-ductal metaplasia (ADM), a reversible transdifferentiation process that may be induced by chronic inflammation, tissue injury, or oncogenic alterations (Marstrand-Daucé et al., 2023; Zheng et al., 2024). Persistent oncogenic signalling promotes progression from ADM to PanIN and ultimately to invasive PDAC, although the coexistence of different precursor lesions and the heterogeneity of pancreatic epithelial populations indicate that PDAC may arise through distinct cellular trajectories (Zhao et al., 2023; Li and Xie, 2022). This progression is accompanied by the accumulation of recurrent genetic alterations, particularly involving KRAS, CDKN2A, TP53, and SMAD4, which regulate cell proliferation, genomic stability, apoptosis, and tumor progression (Huang et al., 2021; Lee et al., 2019; Ferreira et al., 2017). In parallel, the relatively low proportion of neoplastic cells within PDAC tissues can complicate molecular characterization and the identification of tumor-specific alterations (Abdelrahim et al., 2024). As illustrated in Figure 1, the progression from acinar cells through ADM and PanIN to invasive carcinoma is accompanied by the establishment of a dense desmoplastic stroma enriched in CAFs, immune cells, and ECM components. Clinically, PDAC frequently disseminates to distant organs, particularly to the liver, and specific molecular alterations, including SMAD4 inactivation, have been associated with distinct patterns of disease recurrence and metastatic spread (Siegel et al., 2022; Groot et al., 2018). The complex interplay between tumor architecture, molecular alterations such as SMAD4 inactivation, and extensive stromal remodeling provides the biological basis for the profound metabolic reprogramming discussed in the following section.

**FIGURE 1:**
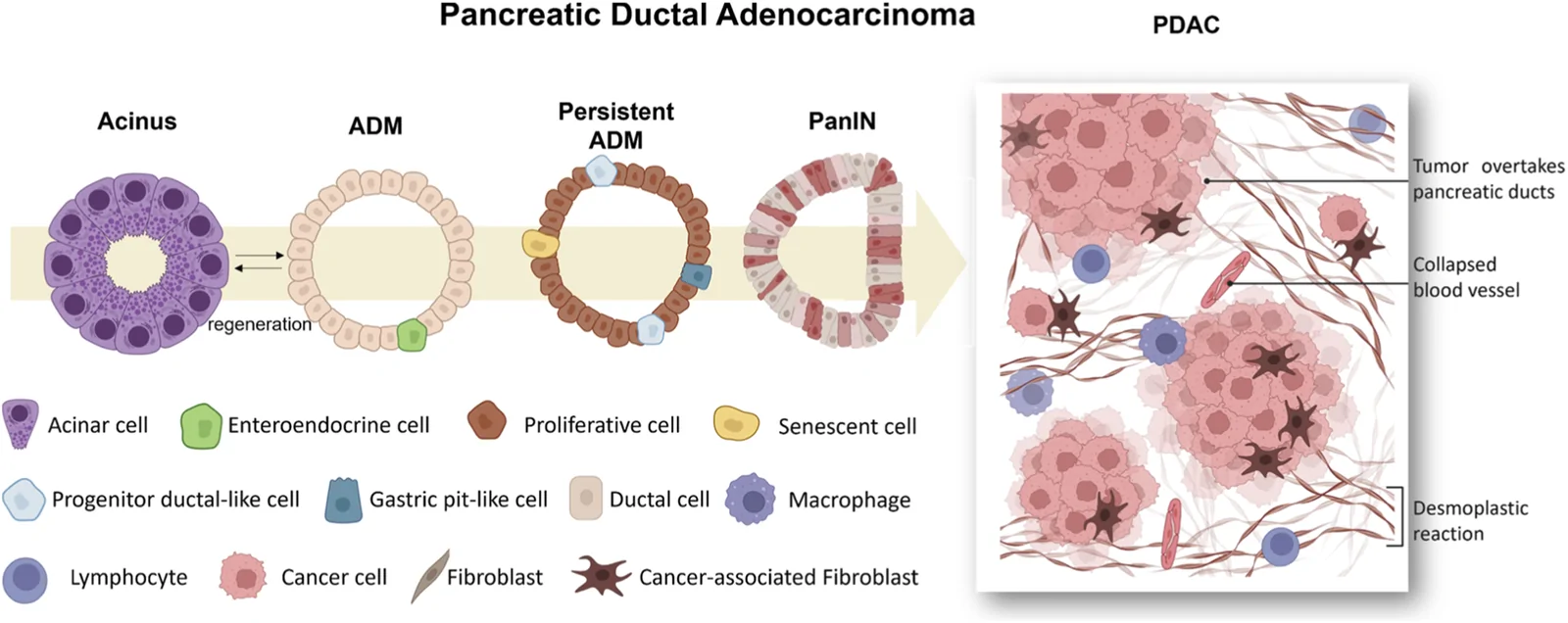
Progression from acinar cells to pancreatic ductal adenocarcinoma (PDAC). Chronic inflammation or oncogenic mutations trigger acinar-to-ductal metaplasia (ADM), which can progress through pancreatic intraepithelial neoplasia (PanINs) to invasive PDAC. The resulting tumor is characterized by malignant epithelial cells embedded in a dense desmoplastic stroma rich in CAFs, immune cells, and ECM components.

## 3 Tumor microenvironment hallmarks

PDAC is among the most stroma-rich solid tumors, with the TME accounting for up to 90% of the tumor mass. This unique histopathological feature reflects a profound cellular and molecular heterogeneity that shapes tumor behavior, immune evasion, and therapeutic resistance (Sherman and Beatty, 2023). The PDAC TME comprises a complex network of neoplastic, stromal, vascular, immune, and neuronal cells embedded in a dense ECM that provides both structural and biochemical support (Hessmann et al., 2020). A defining hallmark of PDAC is desmoplasia, a fibrotic reaction characterized by the excessive deposition of ECM components such as collagen I, fibronectin, proteoglycans, and hyaluronic acid (HA) (Hessmann et al., 2020). The accumulation of HA and cross-linked collagen fibers increases tissue stiffness and interstitial fluid pressure, leading to vascular collapse and impaired perfusion. This biomechanical remodeling generates physical barriers to drug delivery and restricts oxygen diffusion, thereby reinforcing tumor hypoxia. Moreover, ECM remodeling is not a static process, but it is dynamically regulated by stromal enzymes, including matrix metalloproteinases (MMPs), tissue inhibitors of MMPs (TIMPs), and lysyl oxidase-like proteins (LOXLs), which collectively modulate the viscoelastic properties of the TME and influence cell signaling (Elyada et al., 2019). The PDAC stroma contains multiple cell types that engage in extensive paracrine communication. CAFs represent a heterogeneous population with both tumor-promoting and tumor-restraining phenotypes. Distinct CAF subtypes, including inflammatory CAFs (iCAFs), myofibroblastic CAFs (myCAFs), and antigen-presenting CAFs (apCAFs), secrete cytokines, growth factors, and ECM proteins that regulate tumor growth, immune cell recruitment, and matrix organization (Elyada et al., 2019). Immune cells, primarily tumor-associated macrophages (TAMs), neutrophils, and regulatory T cells, dominate the non-epithelial compartment and sustain an immunosuppressive milieu. TAMs polarize toward an M2-like phenotype, secreting IL-10, TGF-β, and VEGF, which suppress cytotoxic T-cell activity and promote angiogenesis. Myeloid-derived suppressor cells (MDSCs) further inhibit adaptive immunity and foster tolerance to tumor antigens. Meanwhile, endothelial cells and neurons participate in reciprocal signaling with tumor and stromal cells, contributing to neurogenic inflammation and perineural invasion, two hallmark features of PDAC progression (Seton-Rogers, 2019). This intricate tumor-stroma crosstalk results in a self-reinforcing feedback loop where tumor cells instruct stromal remodeling, and the remodeled stroma, in turn, sustains cancer cell survival, metabolic adaptation, and resistance to therapy. Due to vascular compression and inefficient perfusion, PDAC tissues are profoundly hypoxic, with oxygen tensions often below 1%. Hypoxia stabilizes hypoxia-inducible factor 1-alpha (HIF-1α), which orchestrates transcriptional programs that promote angiogenesis, glycolysis, and survival (Bogdanov et al., 2022; Czaplinska et al., 2023; Li et al., 2021). Cancer cells respond by shifting from oxidative phosphorylation to glycolysis, even under normoxic conditions, a phenomenon known as the Warburg effect (Liberti and Locasale, 2016). This metabolic reprogramming enables rapid ATP generation and the production of biosynthetic intermediates necessary for proliferation, but it also results in the accumulation of lactate and protons (H^+^), leading to extracellular acidification. The acidic microenvironment further modifies cancer cell behavior, enhancing invasiveness, angiogenesis, and epithelial-to-mesenchymal transition (EMT) (De La Cruz-López et al., 2019). Beyond these effects on tumor cell metabolism and behavior, hypoxia also exerts profound effects on the broader tumor microenvironment, particularly by reshaping the immune landscape of PDAC. It impairs cytotoxic T-cell function, promotes regulatory T-cell activity, and favours the recruitment and persistence of immunosuppressive myeloid populations, thereby contributing to immune evasion and therapeutic resistance (Roerden and Spranger, 2025). In PDAC, more than 90% of glucose-derived carbon is converted into lactate, which is exported through monocarboxylate transporters (MCT1/4). These transporters, together with Na^+^/H^+^ exchangers (NHE-1) and bicarbonate transporters, sustain proton efflux and maintain intracellular pH homeostasis (Felmlee et al., 2020; Greco et al., 2021). The interplay between lactate metabolism and ECM remodeling establishes metabolic symbiosis between stromal and cancer cells; CAFs and macrophages can recycle lactate as an energy substrate, thereby supporting tumor growth under nutrient limitation. Moreover, lactate acts as an important immunomodulatory metabolite by promoting M2-like macrophage polarization, suppressing cytotoxic T-cell activation, and reinforcing the establishment of an immunosuppressive TME (Colegio et al., 2014). Importantly, hypoxia, extracellular acidosis, and lactate accumulation should not be regarded as independent hallmarks of the PDAC microenvironment, but rather as interconnected consequences of metabolic reprogramming that reinforce one another through a self-sustaining feedback loop. Hypoxia promotes HIF-1α-dependent glycolytic reprogramming, increasing glucose consumption and lactate production, while the co-export of lactate and protons further acidifies the extracellular space. In turn, extracellular acidosis enhances EMT, stemness, and reduced drug uptake, whereas lactate functions as both a metabolic substrate and a signaling molecule that promotes CAF activation, immune suppression, and metabolic symbiosis within the TME. Together, these interconnected processes establish a metabolically adaptive microenvironment that sustains tumor progression and contributes to the development of chemoresistance, providing the biological rationale for simultaneously monitoring pH, oxygen, and lactate in advanced 3D PDAC models (Bogdanov et al., 2022; Czaplinska et al., 2023; Li et al., 2021; Liberti and Locasale, 2016; De La Cruz-López et al., 2019; Felmlee et al., 2020; Greco et al., 2021; Alemanno et al., 2023; Wu et al., 2022). Beyond characterizing tumor-cell metabolism, integrating metabolic sensing technologies with advanced immune-competent 3D PDAC models could enable dynamic correlations between local metabolic alterations and immune-cell behaviour, including macrophage polarization and T-cell dysfunction, thereby providing valuable insights into mechanisms of immune escape and responses to emerging immunotherapies. Theoretical and computational frameworks have begun to formalise these metabolic interdependencies: modelling of metabolic coordination and phase transitions in spatially distributed multi-cellular systems reveals how collective metabolic states emerge from local cell-cell interactions, with direct implications for understanding the metabolic heterogeneity of the PDAC TME (Narayanan et al., 2021). Experimentally, heterogeneity in drug response kinetics within cancer-stroma co-cultures has been quantified using live cell imaging readouts, demonstrating that stromal cells modulate cancer cell chemosensitivity in a spatially and temporally heterogeneous manner (Alemanno et al., 2023). The dysregulated biochemical landscape of the PDAC TME, characterized by hypoxia, acidosis, and lactate accumulation, creates both challenges and opportunities for therapy. For instance, real-time monitoring of pH, oxygen, and lactate gradients using optical or electrochemical sensors could provide crucial insights into tumor metabolism and treatment response. Therapeutic strategies aimed at neutralizing tumor acidity, inhibiting lactate transport (e.g., via MCT blockers) (Wu et al., 2022), or modulating ECM stiffness are being actively investigated as ways to reprogram the TME and enhance drug efficacy. Rather than representing isolated metabolic abnormalities, hypoxia, extracellular acidosis, and lactate accumulation constitute an integrated metabolic network that progressively remodels the PDAC microenvironment and ultimately promotes therapeutic resistance. Altogether, the PDAC TME represents a dynamic ecosystem governed by intricate metabolic, mechanical, and immunological interactions (Figure 2). A deeper and spatiotemporal understanding of these hallmarks will be pivotal for developing integrated therapeutic and sensing approaches capable of overcoming the high resistance mechanisms that define this malignancy.

**FIGURE 2:**
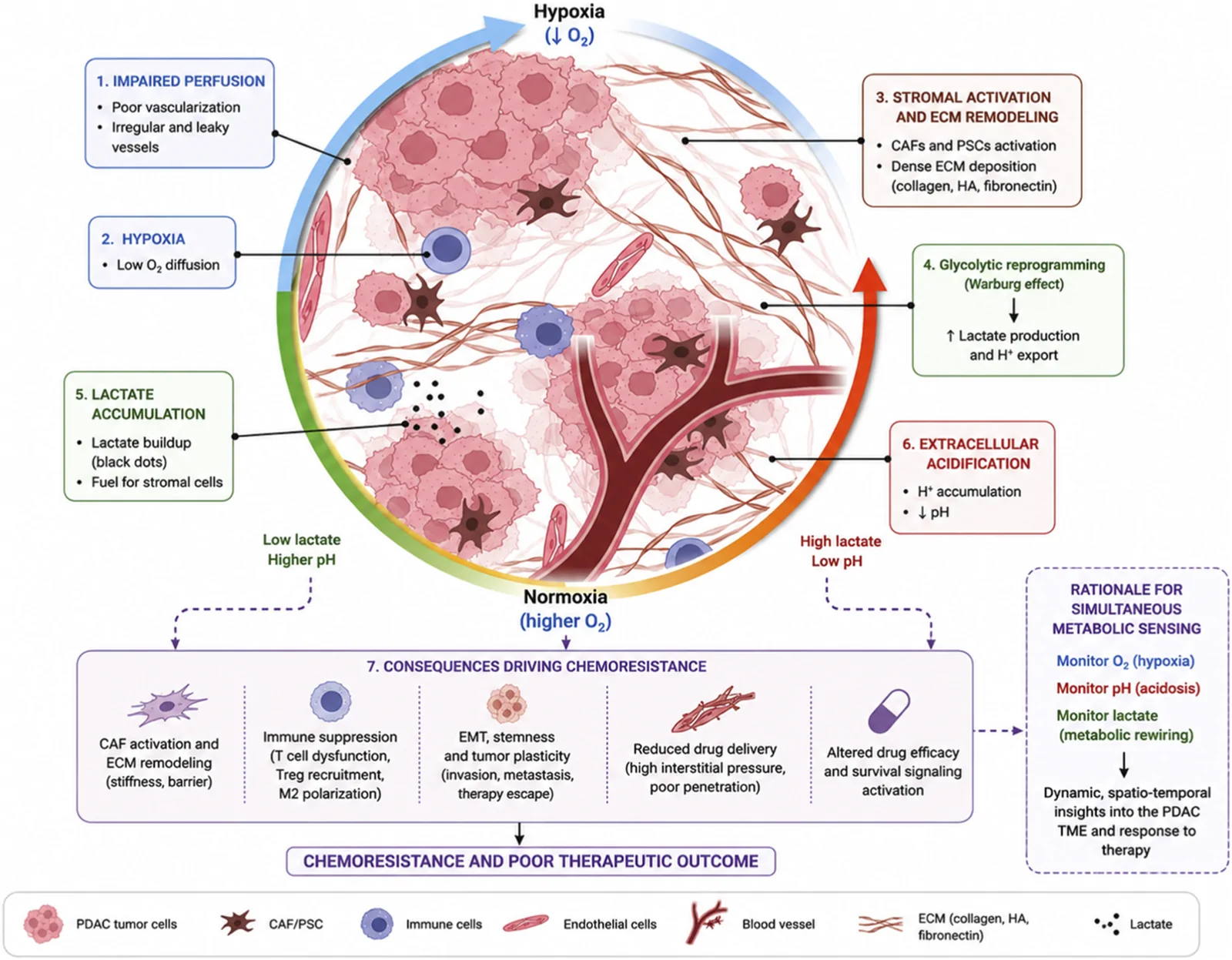
Schematic representation of the interconnected metabolic mechanisms driving chemoresistance in the PDAC tumor microenvironment. The diagram illustrates, through sequential arrows, the progression from impaired vascularization and hypoxia to glycolytic reprogramming, lactate/proton export, extracellular acidification, and downstream stromal and immune effects (CAF activation, EMT, immune suppression), culminating in reduced drug delivery and chemoresistance (see Section 3 for a detailed discussion). The dashed arrows connecting the pH, O_2_, and lactate icons highlight the rationale for their simultaneous monitoring in advanced 3D PDAC models.

## 4 Treatments and chemoresistance in PDAC

PDAC is a highly aggressive malignancy with a dismal prognosis due to its late-stage diagnosis, limited therapeutic options, and marked resistance to conventional therapies (Adamska et al., 2018). Current therapeutic strategies include surgical resection, chemotherapy, radiotherapy, targeted therapy, and immunotherapy, each facing significant challenges in efficacy and resistance (Halbrook et al., 2023). Although these approaches have progressively improved patient management, long-term clinical benefit remains limited due to the intrinsic and acquired resistance of PDAC to treatment (Sie et al., 2023). Chemotherapy therefore remains the cornerstone of PDAC treatment. Gemcitabine has long represented the standard therapeutic option (Albahde et al., 2021; Beutel and Halbrook, 2023), while combination regimens such as FOLFIRINOX and gemcitabine plus nab-paclitaxel have significantly improved survival in selected patient populations, although their efficacy is frequently compromised by severe toxicity and rapid emergence of chemoresistance (Conroy et al., 2018; Zeng et al., 2019; Jung et al., 2023). Radiotherapy, particularly stereotactic body radiotherapy (SBRT), is increasingly used for locally advanced disease (Xiao et al., 2021; Bernard et al., 2020), whereas targeted therapies and immunotherapeutic approaches have shown clinical benefit only in selected molecular subgroups. The principal pharmacological strategies currently available for PDAC are summarized in Table 1. Despite these therapeutic advancements, PDAC remains notoriously resistant to treatment due to the combined contribution of tumor-intrinsic and microenvironment-dependent mechanisms. Altered drug transport mediated by ATP-binding cassette (ABC) transporters, reduced expression of nucleoside transporters, dysregulated drug metabolism, constitutive activation of KRAS signalling, and overexpression of anti-apoptotic proteins collectively reduce the efficacy of conventional chemotherapy (Xiao et al., 2021; Awasthi et al., 2019; Wang et al., 2021; Liang et al., 2017). In parallel, the dense desmoplastic stroma physically limits drug penetration while promoting reciprocal interactions between PSCs, CAFs, immune cells, endothelial cells, and tumor cells, thereby reinforcing therapeutic resistance (Musiu et al., 2024; Wang et al., 2020; Stigliani et al., 2024). Beyond these structural barriers, cell metabolic reprogramming represents another major driver of chemoresistance. Poor vascularization, sustained glycolytic metabolism, and inefficient metabolite clearance generate hypoxic and acidic microenvironment characterized by high extracellular lactate concentrations (Hu and Chen, 2021). These metabolic alterations reduce drug efficacy, promote autophagy, EMT, stem-like phenotypes, and immune suppression, ultimately favouring the selection of therapy-resistant tumor cell populations (Wu et al., 2022; Singh and O’Reilly, 2020). To overcome these mechanisms, several complementary therapeutic strategies are currently under investigation, including modulation of drug transport and apoptosis (Bergonzini et al., 2024; Becker et al., 2024; Seki et al., 2022; Ganji et al., 2022), epigenetic therapies, and more recently, molecularly targeted agents, immunotherapeutic strategies, stromal-targeting approaches, and therapies directed against extracellular acidification and proton transport (Table 1). Although many of these approaches remain under clinical evaluation, they collectively highlight the importance of monitoring the metabolic status of PDAC during treatment. Increasing evidence indicates that metabolic reprogramming is not only a hallmark of chemoresistance but also a therapeutically actionable vulnerability. Pharmacological inhibition of carbonic anhydrase IX (CAIX) with methazolamide was shown to reduce hypoxia-associated acidosis and resensitize gemcitabine-resistant PDAC cells, supporting extracellular pH regulation as a potential strategy to overcome treatment resistance (Sugumar et al., 2025). Similar metabolic-targeting strategies, including HIF-1α inhibition and dual MCT1/MCT4 blockade, have been shown to reverse chemoresistant phenotypes in other cancer models (Dong et al., 2022; Benjamin et al., 2018), and comparable evidence is now emerging specifically in PDAC. Pharmacological inhibition of HIF-1α with PX-478 enhanced the anti-tumor effect of gemcitabine and induced immunogenic cell death in PDAC models, demonstrating that targeting hypoxia-driven signalling can reverse chemoresistant phenotypes directly in pancreatic cancer (Zhao et al., 2015). Lactate transport and glycolytic flux represent a further metabolic vulnerability: pancreatic-stellate-cell-induced gemcitabine resistance in PDAC has been directly linked to LDHA- and MCT4-mediated enhanced glycolysis, indicating that targeting lactate production and transport can sensitize PDAC cells to chemotherapy (Amrutkar et al., 2023). Additional evidence from the NHE1/pH axis further supports extracellular pH regulation as a chemoresistance-reversing strategy in PDAC (Cardone et al., 2015; Pethő et al., 2023). In this context, real-time sensing of extracellular pH, oxygen availability, and lactate accumulation in advanced 3D PDAC models may provide dynamic functional biomarkers of therapeutic response, facilitating treatment evaluation and supporting the development of precision oncology strategies.

**TABLE 1:** Current pharmacological treatments for PDAC with associated chemoresistance mechanisms.

Therapeutic strategy	Drug(s)/Combination	Mechanism of action	Clinical indication	Key mechanisms of chemoresistance	Key references (2023–2025)
Standard Chemotherapy	Gemcitabine	Pyrimidine analog; inhibits DNA synthesis; induces apoptosis	First-line monotherapy for advanced PDAC; also used in adjuvant setting	Drug efflux via P-gp, EMT, dense stromal barrier, KRAS mutations, DNA repair activation	(Huang et al., 2023)
	FOLFIRINOX (5-FU + Leucovorin + Irinotecan + Oxaliplatin	Multi-agent cytotoxic combination targeting DNA replication and repair	Standard first-line regimen for metastatic or locally advanced PDAC	Multi-drug resistance, DNA repair activation, EMT, stromal barrier, cancer stem cells	(Jung et al., 2023)
	Gemcitabine + Nab-Paclitaxel	Microtubule stabilization (nab-paclitaxel) + DNA synthesis inhibition (gemcitabine)	First-line or second-line treatment; improved survival vs. gemcitabine alone	Microtubule alterations, EMT, stromal barrier, drug efflux	(Chen I. M. et al., 2025)
Targeted therapy	Erlotinib	EGFR tyrosine kinase inhibitor	Used in combination with gemcitabine in selected cases (limited benefit)	EGFR pathway mutations, downstream signaling bypass, stromal-mediated resistance	(Liu et al., 2024)
	Olaparib	PARP inhibitor; blocks DNA repair in BRCA1/2-mutated tumors	Maintenance therapy for germline BRCA-mutated metastatic PDAC after platinum response	Restoration of homologous recombination, PARP-independent repair pathway	(M’Baloula et al., 2024)
	Ruxolitinib (Investigational)	JAK1/2 inhibitor; modulates tumor-stroma and immune signaling	Investigational for TME modulation and inflammation-driven PDAC	Activation of alternative JAK/STAT pathways, stromal-mediated drug sequestration	(Deshpande et al., 2025)
	MRTX1133, AMG-510	Selective KRAS G12D inhibitor; blocks oncogenic KRAS signaling	Preclinical and early clinical development for KRAS G12D-mutant PDAC	Adaptive resistance via pathway reactivation, feedback signaling, TME interactions; potential combinatorial strategies with pan-RAS inhibitors	(Wei et al., 2024; Arshad et al., 2023)
Immunotherapy	Pembrolizumab	PD-1 immune checkpoint inhibitor	For MSI-H/dMMR PDAC (∼1%); tissue-agnostic approval	Tumor immune evasion. Low T-cell infiltration, immunosuppressive stroma	(Zhao et al., 2025)
	Nivolumab + Relatlimab (experimental)	Dual PD-1/LAG-3 blockade	Under clinical evaluation for immunoresistant PDAC	Redundant immune checkpoints, immunosuppressive microenvironment	(Grierson et al., 2025)
Stroma-targeting/microenvironmental modulators	PEGPH20	Recombinant hyaluronidase degrading hyaluronic acid in ECM	Increases drug perfusion; studied with chemotherapy	ECM re-synthesis, incomplete stromal depletion, compensatory signaling	(Ko et al., 2023)
	Losartan (repurposed)	Angiotensin II receptor blocker; reduces fibrosis and stromal pressure	Under evaluation as adjuvant to improve vascular perfusion and drug delivery	Alternative fibrosis pathways, stromal heterogeneity	(Ramaswamy et al., 2025)
Metabolic/pH-targeted approaches	CAIX inhibitors (e.g., SLC-0111)	Inhibit carbonic anhydrase IX to reduce extracellular acidification	Preclinical and early clinical studies for PDAC	Alternative pH regulation, metabolic plasticity	(Ghosalkar et al., 2025)
	NHE1 inhibitors (e.g., cariporide, amiloride analogs)	Block Na^+^/H^+^ exchanger; normalize tumor pH and enhance chemosensitivity	Investigational in PDAC preclinical models	Redundant pH-regulating mechanisms, metabolic adaptation	(Sugumar et al., 2025)

## 5 3D *in vitro* PDAC models

Only 6.7% of oncology drugs entering phase I clinical trials ultimately gain FDA approval, a strikingly low success rate largely attributed to poor predictive power of preclinical models, inadequate biodistribution, and unexpected toxicities in patients (Tomás-Bort et al., 2020). The growing recognition of these limitations is also reflected in the FDA Modernization Act 2.0 (2022), which promotes the use of scientifically valid human-based models as complementary approaches to animal testing during preclinical drug development. This regulatory evolution further supports the development of advanced 3D *in vitro* models capable of improving the predictive value of preclinical studies (Han, 2023; Zushin et al., 2023). Traditional preclinical models, including 2D monolayer cultures, animal models, and xenografts, have been invaluable for mechanistic and pharmacological studies, but often fail to accurately reproduce the complex TME of PDAC (Weeber et al., 2017). Their limitations include the absence of native ECM architecture, lack of relevant cell-cell and cell-matrix interactions, and poor replication of the mechanical, chemical, and immune cues that define PDAC biology. These discrepancies contribute to translational failures, as therapeutic responses observed in traditional systems rarely mirror those seen in patients. The roads toward 3D *in vitro* PDAC models have been actively charted, with detailed frameworks describing how cell–cell interactions in three-dimensional pancreatic cancer systems can be engineered and leveraged to better recapitulate disease biology (Delle Cave et al., 2021). To address these challenges, researchers have developed advanced 3D *in vitro* models designed to better replicate the spatial, biochemical, and cellular complexity of the PDAC TME. By integrating tumor cells within natural or synthetic scaffolds, these systems recreate essential aspects of tissue organization, ECM composition, and mechanical stiffness. Tissue engineering approaches further allow the co-culture of multiple cell types, such as CAFs, PSCs, and immune cells, within a physiologically relevant matrix, thereby supporting more accurate modelling of tumor-stroma crosstalk and microenvironmental gradients (Wishart et al., 2021). This cellular and structural complexity is critical, as the dynamic interplay between tumor cells and the ECM drives desmoplasia, drug resistance, and metastatic progression in PDAC (Mitchell et al., 2017). The increasing number of therapeutics targeting the PDAC TME underscores the urgent need for preclinical models that faithfully replicate the patient-specific tumor features to improve drug screening and predict clinical efficacy (Tomás-Bort et al., 2020). In recent years, two major strategies for 3D model development have emerged. The first involves cell-based models, including multicellular spheroids and patient-derived organoids, which can self-organize into structures that preserve key histological and genetic features of primary tumors (Tomás-Bort et al., 2020; Brancato et al., 2020). These models enable studies of tumor heterogeneity, drug penetration, and cell signaling within a context that more closely mimics *in vivo* conditions. The second strategy employs engineering-based systems, such as scaffold-based constructs, 3D bioprinting, and microfluidic tumor-on-chip platforms, which provide fine control over spatial architecture, fluid dynamics, and mechanical cues (Fetah et al., 2019). Tumor-on-chip technologies, in particular, enable dynamic perfusion and real-time analysis of cellular behavior, bridging the gap between static culture systems and living tissue physiology (Monteiro et al., 2025). Collectively, these next-generation 3D *in vitro* models represent a transformative step in PDAC research, offering unprecedented opportunities to investigate tumor biology, therapy resistance, and drug delivery under conditions that better reflect human pathophysiology. Their continued development and standardization will be pivotal for accelerating the translation of preclinical discoveries into clinically effective treatments for pancreatic cancer. The currently available 3D PDAC models differ substantially in their biological complexity, ability to reproduce the tumor microenvironment, compatibility with biosensing technologies, and suitability for different experimental applications. Consequently, no single platform is universally optimal. Table 2 provides a critical comparison of the principal 3D PDAC models currently available, highlighting their strengths, limitations, and suitability for metabolic sensing studies.

**TABLE 2:** Critical comparison of currently available 3D PDAC models for metabolic sensing applications.

Feature	Spheroids	PDOs	Scaffold-based models	3D bioprinted models	Organ-on-chip models
Model design	Self-assembled multicellular aggregates reproducing diffusion-driven tumor heterogeneity. (Monteiro et al., 2025; Totti et al., 2017)	Patient-derived self-organizing structures preserving native tumor architecture and intratumoral heterogeneity. (Roerink et al., 2018; Thorel et al., 2024)	Cells embedded within engineered ECMs with tunable biochemical and mechanical properties. (Antich et al., 2020; LeSavage et al., 2024)	Layer-by-layer biofabrication enabling predefined multicellular architectures. (Li et al., 2020; Sun H. et al., 2025)	Microfluidic multicompartment platforms integrating 3D tissues under controlled perfusion. (Hassell et al., 2017; Haque et al., 2022)
Tumor microenvironment representation	PSCs/CAFs co-culture reproduces desmoplastic interactions but lacks vascularization and immune components. (Domenichini et al., 2019; Norberg et al., 2020)	Preserves patient-specific tumor features, whereas stromal, vascular and immune compartments require dedicated co-culture strategies. (Fan et al., 2019; Krieger et al., 2021)	Enables controlled reconstruction of tumor–ECM–stroma interactions and matrix remodeling. (LeSavage et al., 2024; Gupta et al., 2024)	Spatially organizes tumor, stromal and endothelial compartments within defined architectures. (Sun H. et al., 2025; Li C. et al., 2024)	Integrates tumor, stromal, endothelial and immune compartments under dynamic perfusion. (Haque et al., 2022; Deipenbrock et al., 2025)
Metabolic microenvironment	Naturally develops oxygen, nutrient and extracellular pH gradients through diffusion. (Totti et al., 2017; Cavo et al., 2020a)	Metabolic heterogeneity depends on organoid size and ECM composition. (Roerink et al., 2018; Krieger et al., 2021)	ECM mechanics and composition regulate metabolic adaptation, drug penetration and chemoresistance. (LeSavage et al., 2024; Gupta et al., 2024)	Biofabrication enables engineering of metabolically heterogeneous tissue regions. (Angelopoulos et al., 2020)	Controlled perfusion regulates oxygen, nutrient and metabolite gradients in real time. (Hassell et al., 2017)
Compatibility with metabolic sensing	Supports optical pH/O_2_ sensing and fluorescence-based longitudinal monitoring. (Gupta et al., 2024)	Enables patient-specific metabolic profiling, although optical interrogation may be limited in larger organoids. (Thorel et al., 2024; Krieger et al., 2021)	Supports integration of embedded optical biosensors and sensing hydrogels. (Rizzo et al., 2023; Moldero et al., 2020)	Enables spatial positioning of sensing elements during construct fabrication. (Li et al., 2020)	Integrates continuous optical and electrochemical sensing under physiological flow. (Turetta et al., 2018)
Primary applications	High-throughput drug screening, metabolic profiling and therapy-response monitoring. (Delle Cave et al., 2021; Gupta et al., 2024)	Precision medicine, patient stratification and prediction of therapeutic response. (Krieger et al., 2021)	Tumor–ECM interactions, stromal biology and matrix-mediated chemoresistance. (LeSavage et al., 2024; Gupta et al., 2024; Rizzo et al., 2023; Moldero et al., 2020)	Multicellular tissue engineering and spatial drug-response studies. (Li M. et al., 2024)	Dynamic drug testing, vascular biology and longitudinal metabolic monitoring. (Adnan et al., 2026)
Major advantages	Simple, reproducible, scalable and cost-effective. (Totti et al., 2017; Cavo et al., 2020a)	Highest patient specificity and clinical relevance. (Thorel et al., 2024)	Highly controllable biochemical and mechanical microenvironment. (Antich et al., 2020)	Precise spatial control of cells, biomaterials and tissue architecture. (Li et al., 2020)	Closest approximation to the native PDAC microenvironment. (Hassell et al., 2017)
Key limitations	Limited vascularization, tissue compartmentalization and stromal complexity. (Cavo et al., 2020a; Domenichini et al., 2019)	Matrigel dependence and incomplete representation of stromal and immune components. (Fan et al., 2019)	Biomaterial variability and matrix optimization may affect reproducibility. (Antich et al., 2020; Hoshiba, 2017)	Specialized equipment, bioink optimization and relatively low throughput. (Li et al., 2020; Li M. et al., 2024)	High technical complexity, limited standardization and reduced scalability. (Hassell et al., 2017; Turetta et al., 2018)

### 5.1 Spheroids

Spheroids represent one of the most accessible and widely used 3D *in vitro* models for studying tumor biology. These multicellular aggregates, typically grown in suspension, can self-assemble from cancer cells alone or in combination with stromal components. Incorporating natural peptides or matrices such as collagen or Matrigel enhances cell-cell adhesion and ECM deposition, allowing spheroids to develop tissue-like structures with more physiologically relevant spatial organization and gene expression profiles compared to 2D cultures (Totti et al., 2017). Importantly, spheroids can also recreate key microenvironmental features of solid tumors, including the formation of hypoxic and acidic cores, nutrient and oxygen gradients, and heterogeneous proliferation zones, which are all hallmark characteristics of PDAC (Cavo et al., 2020a). These features make spheroids a particularly valuable platform for modelling PDAC, a cancer marked by dense desmoplasia, hypoxia, and profound cellular heterogeneity. Indeed, PDAC spheroids demonstrate greater resistance to chemotherapy and radiotherapy than conventional monolayer cultures, reflecting the *in vivo* drug resistance observed in patients (Wishart et al., 2021; Domenichini et al., 2019). Such chemoresistance has been linked to the emergence of cancer stem-like cell populations within spheroids, which display enhanced survival capacity and reduced metabolic activity, mimicking the tumor’s adaptive responses to metabolic stress. To further improve physiological relevance, recent PDAC spheroid models have evolved toward heterotypic co-culture systems, where cancer cells are combined with stromal components such as PSCs or CAFs. Among these, PSC-containing models are particularly valuable because they closely reproduce the desmoplastic microenvironment of PDAC and enable the investigation of tumor–stroma metabolic crosstalk, ECM remodeling, hypoxia-driven chemoresistance, and stromal-mediated therapeutic resistance. Upon direct contact with tumor cells, PSCs differentiate into activated CAFs, contributing to ECM remodeling and promoting a fibrotic, mechanically stressed microenvironment (Norberg et al., 2020). For example, Struth et al. (Struth et al., 2024) developed a 3D co-culture spheroid model that captures the biomechanical interplay between PDAC cells and fibroblasts. Their imaging studies revealed that fibroblast contractility and tumor-stroma compaction occur only when fibroblasts can physically adhere to PDAC nodules, leading to the formation of densely packed, desmoplastic spheroids (Figure 3a). Interestingly, co-cultures with MRC5 fibroblasts exhibited stronger contractility and aggregation than those with PSCs, indicating that juxtacrine signaling and myofibroblastic activation play key roles in mediating stromal mechanics. Such models provide a powerful experimental platform to investigate PDAC desmoplasia, mechanical stress, and drug diffusion in fibrotic tumors. Overall, these findings highlight that incorporating PSCs into spheroid models substantially enhances their ability to reproduce the desmoplastic and metabolically active PDAC microenvironment, thereby providing a more physiologically relevant platform for investigating tumor–stroma interactions, metabolic remodeling, chemoresistance, and therapeutic response. By recapitulating the biophysical and biochemical complexity of the PDAC TME, spheroid-based systems bridge the gap between oversimplified 2D assays and more costly organoid or animal models, offering an intermediate yet highly informative tool for translational cancer research.

**FIGURE 3:**
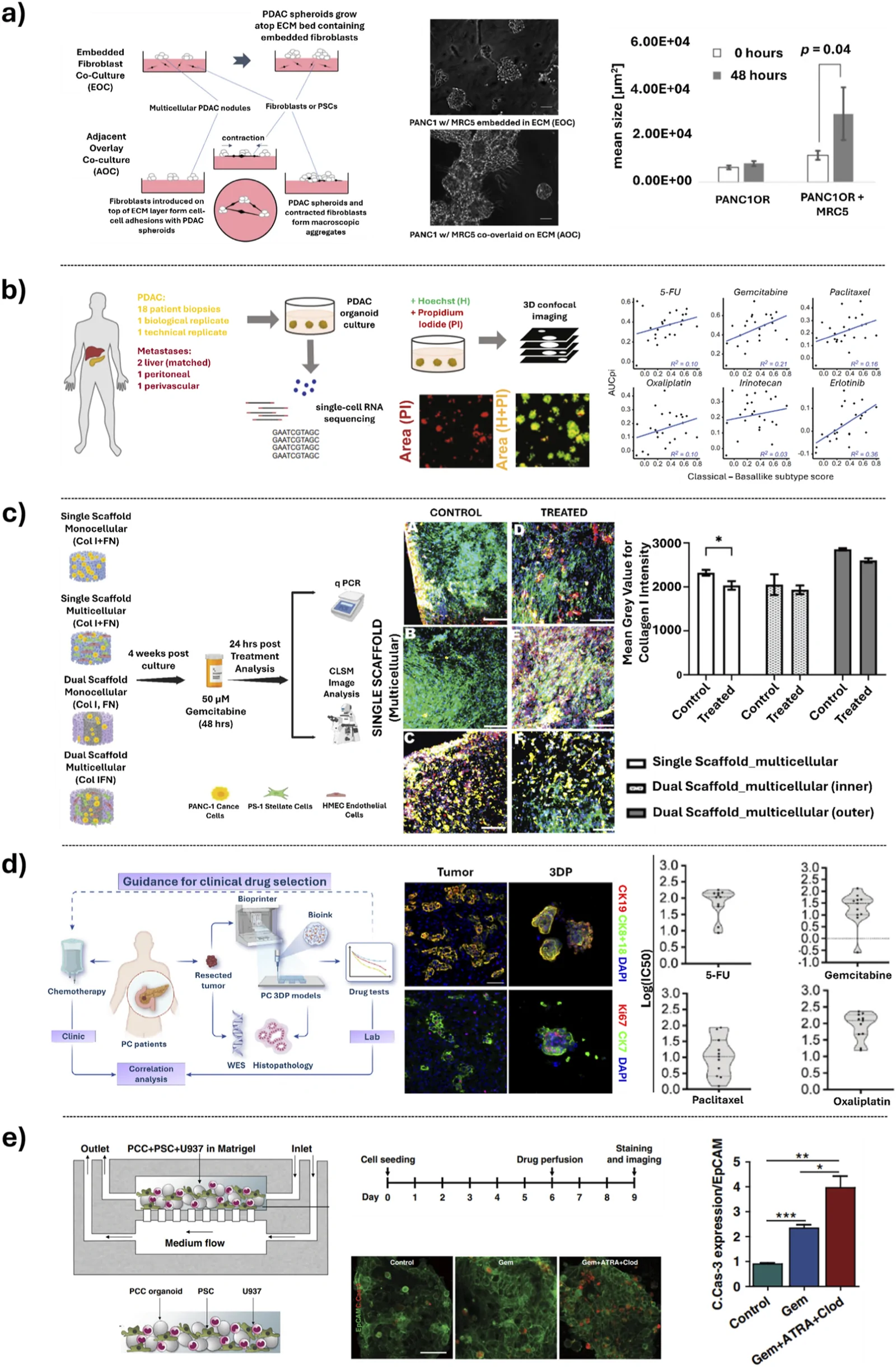
Representative examples of 3D *in vitro* PDAC models. **(a)** Co-culture spheroid model capturing the biomechanical interplay between PDAC cells and fibroblasts, illustrating desmoplastic compaction and stromal contractility. Reproduced from Struth et al. (2024) under the terms of the CC BY-NC-ND 4.0 license. **(b)** Patient-derived PDAC organoids used for single-cell transcriptomic profiling and imaging-based drug screening, revealing intratumoral heterogeneity and subtype-dependent drug sensitivity. Adopted from Krieger et al. (2021) under the terms of the Creative Commons Attribution License (CC BY 4.0). **(c)** 3D porous scaffold-based PDAC model (e.g., collagen-functionalized or ECM-mimetic matrices) integrating pancreatic cancer cells with activated pancreatic stellate cells in spatially controlled configurations. Adopted from Gupta et al. (2024) under the terms of the Creative Commons Attribution License (CC BY 4.0). **(d)** A patient-derived, extrusion-based 3D bioprinted PDAC model with spatially controlled tumor–stroma architecture, using GelMA-based ECM-mimetic bioinks to organize pancreatic cancer cells and stromal fibroblasts within defined compartments. Reproduced from Sun H. et al. (2025) under the terms of the CC BY-NC-ND 4.0 license. **(e)** Microfluidic PDAC-on-a-chip integrating patient-derived organoids to model tumor-stroma bidirectional interactions and evaluate stromal-targeting therapeutic strategies. Adopted from Haque et al. (2022) under the terms of the Creative Commons Attribution License (CC BY 4.0).

### 5.2 Organoids

Organoids are advanced three-dimensional tissue models that self-organize into structures resembling native organs in both architecture and function. Unlike spheroids, which arise from multicellular aggregation, organoids originate from single cells, typically induced pluripotent stem cells (iPSCs), adult stem cells, or primary tumor cells, embedded within a supportive hydrogel matrix, such as Matrigel. Within this biomimetic microenvironment, cells proliferate and differentiate through self-directed morphogenetic programs, giving rise to complex multicellular assemblies that closely recapitulate the structural and functional hierarchy of *in vivo* tissues (Roerink et al., 2018). In cancer research, organoids have emerged as powerful tools for modelling tumor initiation, progression, and therapeutic response. They are generally classified into two main categories (Fan et al., 2019). Engineered cancer organoids (ECOs) are derived from normal tissue organoids generated from iPSCs or adult stem cells and subsequently genetically modified to introduce specific oncogenic mutations, enabling controlled studies of tumorigenesis. In contrast, PDOs are established directly from primary tumor samples, biopsies, or circulating tumor cells, preserving the genetic, phenotypic, and histopathological heterogeneity of the original malignancy. Owing to their rapid generation and high fidelity to patient-specific tumor biology, PDOs have become particularly valuable for drug screening, biomarker discovery, and precision oncology (Thorel et al., 2024; Karakasheva et al., 2021). In the context of PDAC, organoid technology has proven instrumental for dissecting intratumoral heterogeneity and drug resistance mechanisms. Krieger et al. (Krieger et al., 2021) established organoids from 18 primary PDAC tumors and two matched liver metastases to investigate cell-state diversity using single-cell transcriptomic analysis (Figure 3b). The study revealed multiple conserved tumor cell states across patients, including a proliferative progenitor state and a differentiated secretory state, organized along a developmental hierarchy culminating in “classical” epithelial cells. Notably, ‘classical’ and ‘basal-like’ subtypes coexisted within individual organoids, reflecting the high intratumoral heterogeneity. An imaging-based drug screen further demonstrated that expression of ‘classical’ subtype genes correlated with enhanced drug sensitivity, suggesting that tumor cell composition profoundly influences treatment response. Despite these advantages, conventional PDOs still present several limitations that should be considered when selecting the most appropriate experimental model. During prolonged culture, stromal, vascular, and immune components are progressively depleted, reducing the ability of PDOs to fully recapitulate the complexity of the native PDAC tumor microenvironment. In addition, the use of exogenous extracellular matrix formulations and differences in culture protocols may affect reproducibility across laboratories, while prolonged expansion may result in clonal selection and genetic or phenotypic drift. Furthermore, the lack of a functional vascular network limits the establishment of physiological oxygen and nutrient gradients. To overcome these limitations, several PDO-based strategies are currently being explored, including multicellular co-culture systems to restore stromal interactions and engineered extracellular matrices to improve reproducibility and provide greater control over the biochemical and mechanical properties of the culture microenvironment (Schuth et al., 2022; Lumibao et al., 2024; Li C. et al., 2024). Overall, PDAC organoids remain among the most promising platforms for precision oncology. Continued efforts to preserve the multicellular complexity of the TME while improving reproducibility are expected to further enhance their predictive value for patient-specific drug screening and translational applications.

### 5.3 Scaffold-based models

Scaffold-based culture models represent an engineering-driven approach to reproduce the three-dimensional organization and biophysical context of 3D tumors. In this setting, a *scaffold* refers to a synthetic or natural biomimetic structure that provides the mechanical support and biochemical cues necessary to guide cell growth, differentiation, and communication (Liu and Vunjak-Novakovic, 2016). Despite the widespread use of the term, “scaffold” encompasses a broad range of materials with diverse physicochemical properties. These include porous ceramic or polymeric structures made from synthetic polymers such as poly-lactic acid (PLA), poly (lactic-co-glycolic acid) (PLGA), poly (L-lactic acid) (PLLA), and poly (caprolactone) (PCL) (Knutson et al., 2017). Moreover, hydrogels, crosslinked polymeric networks of artificial or natural origin, offer highly tunable viscoelasticity and biochemical functionality. Common examples include collagen, fibrin, alginate, chitosan, gelatin, HA, and silk fibroin (Antich et al., 2020; Sapudom and Pompe, 2018). Recently, tissue-derived biomaterials like Matrigel and decellularized extracellular matrix (dECM) have gained popularity in cancer research due to their rich composition of native proteins and matrix-bound signaling molecules (Hoshiba, 2019). However, their biological origin introduces several limitations, including high batch-to-batch variability, elevated costs, and complex purification processes, which hinder reproducibility in drug testing and therapeutic development (Hoshiba, 2017). To address these limitations, LeSavage et al. (LeSavage et al., 2024) developed a tunable hyaluronic acid–elastin-like protein (HELP) hydrogel platform, enabling modulation of matrix stiffness and demonstrating that increased rigidity promotes chemoresistance in PDAC organoids via CD44-mediated signaling and enhanced drug efflux. A complementary approach was reported by Gupta et al. (Gupta et al., 2024), who developed a 3D porous scaffold-based PDAC model integrating pancreatic cancer cells with activated pancreatic stellate cells in spatially controlled configurations (Figure 3c). In this system, cells were seeded within biocompatible porous polymeric scaffolds (e.g., collagen-functionalized or ECM-mimetic matrices) that support cell infiltration and ECM deposition, enabling long-term culture and stromal activation. By comparing mono- and co-culture conditions, as well as different tumor–stroma spatial arrangements, the authors demonstrated that stellate cell activation drives ECM remodeling and increases matrix density, which in turn significantly reduces gemcitabine penetration and efficacy. Importantly, spatial compartmentalization of tumor and stromal components resulted in significantly higher chemoresistance compared to mixed cultures, highlighting the critical role of tissue architecture in regulating therapeutic response. Collectively, scaffold-based models offer a powerful and tunable platform for studying the reciprocal interactions between cancer cells and their ECM. By integrating biomaterial engineering with PDAC biology, these systems provide new opportunities to dissect mechanoresponsive pathways and to identify strategies aimed at overcoming stroma-mediated therapeutic resistance. A further dimension is added when scaffolds are coupled with integrated optical biosensors: pH-sensing hybrid hydrogels embedded within 3D tumor spheroid cultures enable real-time, non-invasive monitoring of metabolic responses to drug treatment, allowing direct correlation between ECM composition, mechanical state, and extracellular acidification (Rizzo et al., 2023). Similarly, pH-sensing microparticles incorporated into additive-manufactured scaffolds have been used to probe the pH microenvironment of mesenchymal stromal cell cultures, demonstrating spatial heterogeneity in extracellular acidity that mirrors the gradients observed in tumor tissues (Moldero et al., 2020). These sensing-integrated scaffold strategies represent a convergence of tissue engineering and metabolic monitoring that is directly applicable to PDAC research. Despite these advantages, scaffold-based systems present specific limitations relative to other 3D platforms. Compared with spheroids, they require more elaborate fabrication and quality-control steps, and batch-to-batch variability in biomaterial composition and stiffness, particularly for tissue-derived matrices, can compromise reproducibility across laboratories. Unlike organ-on-chip systems, most scaffold-based cultures remain static and lack active perfusion, limiting long-term nutrient and oxygen renewal. Nonetheless, their high tunability of stiffness, composition, and embedded sensor placement makes them particularly well suited for mechanistic studies isolating the specific contribution of ECM properties to metabolic sensing readouts (Table 2).

### 5.4 3D bioprinted models

3D bioprinting was developed to overcome the limitations of conventional scaffold-based approaches by providing precise spatial control over the deposition of cells and biomaterials within three-dimensional constructs (Li et al., 2020). This technology enables the fabrication of tissue-mimetic architectures that more accurately reproduce the structural complexity and cellular heterogeneity of native tumors. Various bioprinting techniques have been established in recent years, including stereolithography-based bioprinting (Miri et al., 2018), extrusion-based bioprinting, inkjet-based bioprinting (Angelopoulos et al., 2020), laser-assisted bioprinting, and electrospinning-based bioprinting (Chen et al., 2018). Regardless of the printing strategy, these techniques rely on computer-aided design (CAD) to deposit bioinks, mixtures of cells, hydrogels, and signaling molecules, in precise spatial patterns, allowing controlled tissue organization and composition. A recent study (Sun H. et al., 2025) introduced a patient-derived, extrusion-based 3D bioprinted PDAC model with spatially controlled tumor–stroma architecture, using GelMA-based ECM-mimetic bioinks to organize pancreatic cancer cells and stromal fibroblasts within defined compartments (Figure 3d). The use of patient-derived tumor and stromal cells significantly enhances the physiological and translational relevance of the system compared to conventional cell-line-based models. The construct reproduces a heterogeneous “clustered” tumor organization embedded within a stromal matrix, enabling the study of spatially regulated tumor–stroma interactions and ECM remodeling. Functionally, the model recapitulates key PDAC features, including enhanced stromal activation, increased collagen-rich ECM deposition, and formation of diffusion-limited microregions, which collectively contribute to a more chemoresistant phenotype. Compared to less organized configurations, the spatially structured bioprinted constructs exhibit stronger pro-tumoral stromal signaling and reduced sensitivity to standard therapies, reflecting impaired drug penetration and microenvironment-driven resistance. Overall, the study demonstrates that patient-derived cellular composition, ECM composition, and spatial architecture synergistically regulate tumor aggressiveness and therapeutic response. Earlier work by Li et al. (Li M. et al., 2024) proposed a “sea-and-island” PDAC tumor construct, in which cancer cell clusters are printed as discrete islands within a fibroblast-populated ECM, capturing heterotypic spatial interactions and metabolic stress responses. By integrating precise spatial control with the ability to reproduce the hierarchical and biochemical complexity of native tissues, 3D bioprinting establishes an advanced platform for modelling PDAC architecture and tumor-stroma reciprocity. This approach enables the investigation of how mechanical cues, metabolic gradients, and stromal dynamics collectively influence tumor progression and treatment response. Electrospun nanofibers represent a complementary fabrication strategy within this landscape: by recapitulating fibrous ECM architecture at the nanoscale, electrospinning-based approaches support the engineering of cancer 3D models that capture matrix-cell interactions with high spatial fidelity, and have shown utility in both cancer modelling and drug delivery applications (Cavo et al., 2020b). Compared with scaffold-based and spheroid cultures, 3D bioprinted constructs offer precise, user-defined spatial positioning of multiple cell types and biomaterials, enabling systematic investigation of how tumor–stroma architecture shapes metabolic gradients and chemoresistance. This spatial control comes at the cost of specialized bioprinting equipment, bioink optimization, and generally lower throughput than spheroid- or organoid-based approaches, which currently limits their routine use for large-scale drug screening. Sensor integration during the printing process also remains technically demanding and largely confined to proof-of-concept studies, in contrast to the more established sensor-embedding protocols already available for scaffold- and hydrogel-based systems (Table 2).

### 5.5 Microfluidic organ-on-a-chip

Microfluidic organ-on-a-chip technologies have revolutionized biological research by enabling dynamic, real-time monitoring of cellular behavior within physiologically relevant environments. Unlike traditional static cultures, these systems integrate advanced imaging and fluidic control to recreate tissue-specific microenvironments under continuous perfusion, offering superior reproducibility and experimental flexibility (Brancato et al., 2020). By incorporating microchannels that mimic vascular networks, organ-on-a-chip platforms ensure a steady supply of oxygen and nutrients while efficiently removing metabolic waste products, thus promoting long-term 3D cell growth and functional maintenance (Hassell et al., 2017). In the context of PDAC, Haque et al. (Haque et al., 2022) developed an orthotopic PDAC-on-a-chip model integrating PDOs to model bidirectional interactions between epithelial and stromal compartments (Figure 3e). This microfluidic system successfully reproduced hallmark features of the PDAC microenvironment, including desmoplastic stroma, immune cell infiltration, and dynamic biochemical gradients. Functional validation demonstrated that stromal-targeting agents significantly enhanced the cytotoxic efficacy of chemotherapeutics within the chip, an effect not observed in conventional monocultures. These findings highlight the potential of PDAC-on-a-chip models as preclinical tools for personalized medicine, offering a bridge between *in vitro* experimentation and patient-specific therapeutic optimization. By capturing the complexity of tumor-stroma crosstalk under perfused and controlled conditions, organ-on-a-chip systems provide a next-generation platform for drug testing, mechanistic studies, and the investigation of tumor adaptation in response to therapy. More broadly, microfluidic platforms have been explored as enabling technologies for personalized cancer medicine, with early frameworks proposing their integration with 3D tumor models for patient-specific drug screening (Turetta et al., 2018; Adnan et al., 2026; Deipenbrock et al., 2025). Organ-on-chip architectures have also been proposed as platforms for immune system modelling, with human immune-system-on-chip designs enabling investigation of immune cell behavior and immunotherapy responses in controlled microenvironments (Polini et al., 2019; Chen et al., 2024; Geyer et al., 2023), a particularly relevant capability given the immunosuppressive TME of PDAC. Among the currently available 3D PDAC platforms, organ-on-chip systems provide the closest approximation to physiological perfusion and the greatest capacity for continuous, longitudinal biosensing. This advantage is counterbalanced by considerable technical complexity: microfabrication, multicellular loading, and long-term maintenance of perfused cultures require specialized expertise, resulting in lower throughput and higher cost than spheroid- or scaffold-based models, and limited standardization across laboratories remains an obstacle to their broader adoption for routine metabolic sensing studies (Table 2).

## 6 Emerging sensing strategies for characterizing the PDAC microenvironment

### 6.1 Measuring extracellular and intracellular pH fluctuations

PDAC cells exhibit a reversed pH gradient compared to normal cells, characterized by an alkaline intracellular pH (pH_i_) and an acidic extracellular pH (pH_e_) (Wood et al., 2022). This dysregulated acid-base balance is sustained by the upregulation of proton and lactate transporters that maintain pH homeostasis (Dahl-Jensen et al., 2018). The active extrusion of H^+^ not only alkalinizes the cytoplasm but also reduces CO_2_ production through inhibition of the tricarboxylic acid (TCA) cycle and oxidative phosphorylation (OXPHOS) (Koltai, 2017; Corbet and Feron, 2017). Carbonic anhydrases (CAs) further modulate pH by catalyzing the reversible hydration of CO_2_, supporting pH_i_ alkalinization and extracellular acidification (Becker and Deitmer, 2020). Under physiological conditions, pancreatic ductal cells secrete an alkaline (pH ∼8–8.5) NaHCO_3_-rich fluid to neutralize gastric acid, resulting in cyclical pH fluctuations across the apical and basolateral membranes (Dahl-Jensen et al., 2018; Di Molfetta et al., 2023). These oscillations are balanced by ion transporters such as SLC26, NHE-1, NBCs, and H^+^/Na^+^ ATPases, which tightly regulate pH_i_ (Schnipper et al., 2020; Jun et al. 2017). In PDAC, this equilibrium is profoundly disrupted: acid extruders such as MCT4 and NHE-1 are overexpressed, contributing to the acidic pH_e_ (6.4–7.0) and elevated pH_i_ observed in tumor cells (Cardone et al., 2019). The acidic TME induces apoptosis in normal cells but enhances survival, proliferation, and metastatic potential in malignant ones (Pillai et al., 2019). Moreover, PDAC cells, adapted to the pancreas’s natural pH fluctuations, leverage this environment to drive immune evasion, chemoresistance, and metastatic dissemination (Cappellesso et al., 2022; Sahin et al., 2018). Extracellular acidosis further promotes the emergence of cancer stem cells (CSCs), endowed with self-renewal capacity and high drug resistance (DiNatale et al., 2022; Zhou et al., 2021; Peppicelli et al., 2017). Overall, the reversal of the pH gradient in PDAC is a hallmark of metabolic reprogramming and malignancy (Zheng et al., 2020; Persi et al., 2018). To characterize these phenomena, accurate measurement of intra- and extracellular pH is essential. Since pH varies across cellular compartments, ranging from 8.0 in mitochondria to 5.0 in lysosomes (Flinck et al., 2018), several biosensing technologies have been developed. Traditional pH electrodes, while precise, are unsuitable for cellular-scale studies due to their invasive design (Grasso et al., 2023). Electrochemical methods, such as potentiometric ion-selective electrodes (ISEs), Ion-Sensitive Field-Effect Transistors (ISFETs), and Electrolyte-Gated Field-Effect Transistors (EGFETs), enable high-sensitivity pH_e_ monitoring (Ghoneim et al., 2019). However, they face limitations such as sensor instability, voltage drift, and dependence on bulky reference electrodes (Schnipper et al., 2020; Ali, 2017). Complementary imaging approaches, including radionuclide PET imaging, scanning electrochemical microscopy (SECM), and magnetic resonance-based methods, offer non-invasive, real-time mapping of pH heterogeneity within tissues (Steinegger et al., 2020; Genicio et al., 2021). In particular, ^1^H magnetic resonance spectroscopy (^1^H MRS) enhances sensitivity and spatial resolution compared to traditional ^31^P MRS, enabling precise quantification of pH_e_ gradients in tumors (Lutz and Bernard, 2019). These advancements collectively improve our ability to analyze pH-dependent tumor behavior and guide therapeutic interventions targeting acid-based dysregulation. Among modern biosensing technologies, optical pH sensors have gained prominence for their non-invasive, real-time capabilities with high spatial resolution (Hassan Akhtar et al., 2024). These systems exploit fluorescence, absorbance, or luminescence changes in pH-responsive materials. Compared to electrochemical sensors, they are less prone to drift and interference, and can be miniaturized for *in vivo* studies (Steinegger et al., 2020). Ratiometric fluorescence sensors, Förster Resonance Energy Transfer (FRET)-based probes, and near-infrared (NIR) indicators further improve quantitative precision and imaging depth (Hassan Akhtar et al., 2024). Among particle-based optical pH sensors, silica microparticles functionalized with ratiometric fluorescent dyes, combining a pH-sensitive indicator and a reference dye, have proven particularly effective for non-invasive, high-resolution mapping of extracellular pH in living 3D systems. Chandra et al. demonstrated highly sensitive pH microsensors based on pyranine-functionalized silica particles, achieving quantitative ratiometric pH sensing with single-cell resolution in cell cultures (Chandra et al., 2021). The same platform was extended to fully automated computational analysis of organelle acidification, enabling precise quantification of endo-lysosomal pH dynamics at the single-organelle level (Chandra et al., 2022). More recently, modular hybrid silica–hydrogel architectures have been introduced to further improve sensor biocompatibility and pH responsiveness, enabling high-resolution extracellular pH sensing in both tumor and healthy cell models (Iuele et al., 2025). Other published works presented pH-sensing particles as non-invasive metabolic monitoring tools in tumor spheroids and alginate-based 3D models of PDAC, where real-time pH readouts correlated with drug-induced metabolic shifts (Siciliano et al., 2024; Rizzo et al., 2023). Recently, silica-based microparticles loaded with two dyes, a pH-sensitive fluorescein and a reference rhodamine B, have been employed for single-cell level monitoring of pH_e_ in 3D patient-derived PDAC models (Figure 4a) (Forciniti et al., 2026). The use of these ratiometric optical pH sensors enabled the investigation of pH fluctuations in response to chemotherapeutic treatment, providing a robust platform for both high spatiotemporal resolution pH sensing and personalized medicine applications. Kromer et al. (Kromer et al., 2024) also engineered a dual-dye polystyrene nanoparticle sensor, combining a pH-inert reference dye (Nile red) and a fluorescein-based pH indicator, achieving sensitive ratiometric pH_e_ detection at the cell surface (pH 5.5–9.0) (Figure 4b). Following these advances in extracellular pH sensing, optical-based sensors have also been employed for intracellular pH monitoring. For instance, a ratiometric fluorescent probe named Cys-Cys-pH was developed to sequentially detect cysteine and intracellular pH changes in pancreatic cancer cells (Figure 4c). (Qin et al., 2022) This probe undergoes a cysteine-triggered structural transformation that enables pH-dependent fluorescence shift, allowing real-time imaging of intracellular acid-base fluctuations during chemotherapy. Similarly, Ouologuem et al. (Ouologuem et al., 2025) reported silica-based ratiometric optical pH sensors, which displayed a high pH responsiveness and reversibility, enabling real-time quantification of endo-lysosomal acidification through confocal microscopy (Figure 4d). Upon cellular internalization, the sensors shift from yellow to red fluorescence, reflecting confinement in acidic intracellular compartments and allowing precise ratiometric tracking of pH over time. Although not yet applied to *in vitro* PDAC models, this system represents a promising intracellular sensing strategy that could be readily adapted to investigate lysosomal pH dysregulation in pancreatic cancer models. A previous work using fluorescent capsule-based sensors for measuring acidification of endocytic compartments in cells with altered expression of V-ATPase subunit V_1_G_1_ established that endo-lysosomal acidification is tightly linked to V-ATPase activity. Specifically, V-ATPase subunit V_1_G_1_ expression directly modulated the rate and extent of vesicular acidification as quantified by ratiometric optical sensors (De Luca et al., 2015). Beyond pH, the PDAC TME is characterised by dysregulation of multiple ionic species that collectively shape the tumour microenvironment. Ratiometric fluorescent sensors for potassium ions, based on IPG4-functionalised silica microparticles, have recently been demonstrated for selective K^+^ detection and fluorescence imaging in cellular systems (Colella et al., 2024), opening the possibility of multi-analyte microenvironmental monitoring within 3D PDAC models. Similarly, ratiometric organic electrospun fibers have been shown to enable localised and reversible ion sensing with micrometer-scale spatial resolution (Onesto et al., 2023; Del Mercato et al., 2015), providing a fibre-format sensing architecture that could be integrated into 3D tumour constructs for simultaneous pH and ion mapping.

**FIGURE 4:**
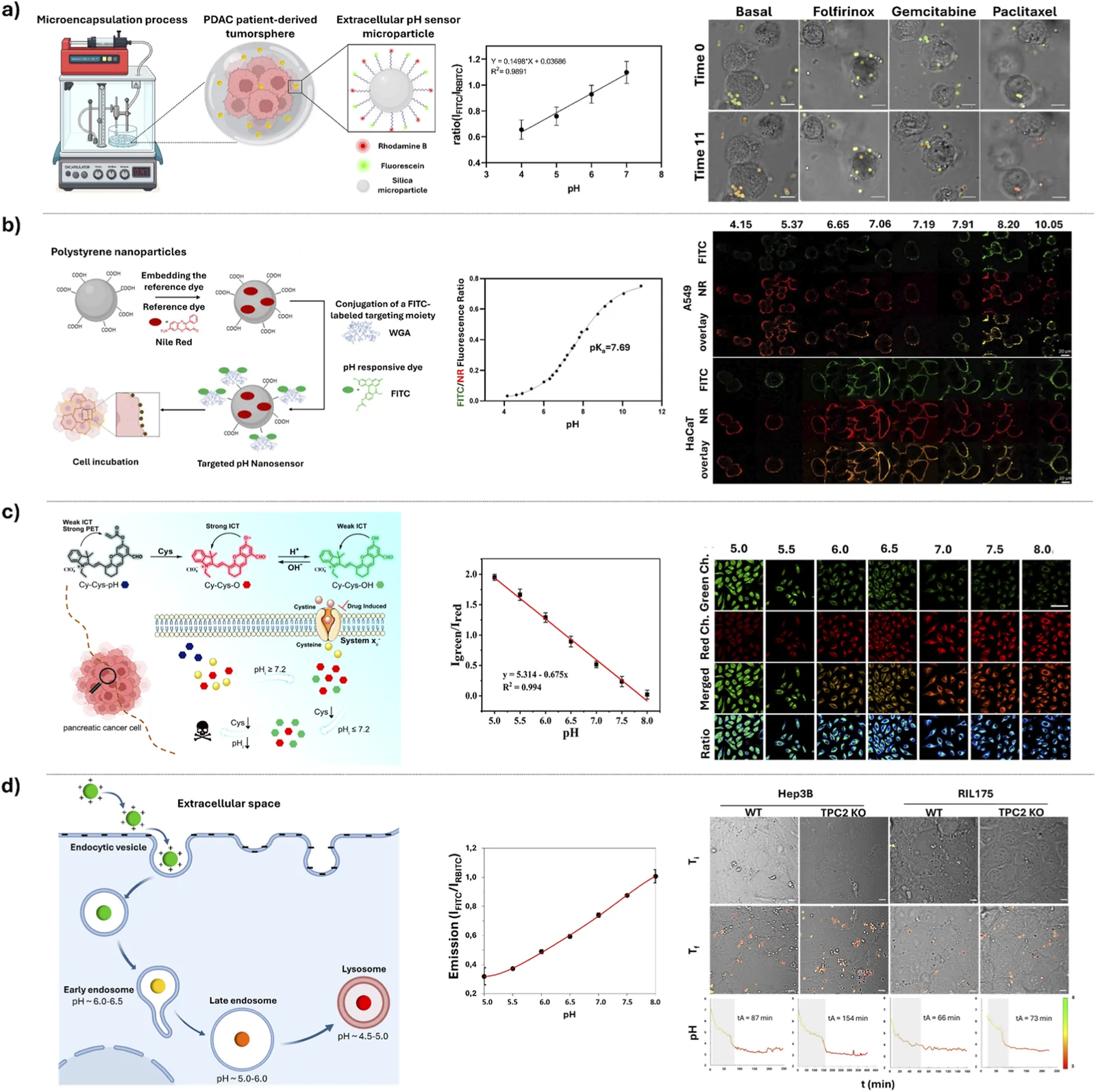
Representative examples of extra- and intracellular optical pH sensing strategies in cancer models. **(a)** Ratiometric silica-based microparticles functionalized with a pH-sensitive dye (fluorescein) and a reference dye (Rhodamine B) were co-encapsulated with patient-derived PDAC cells in alginate microgels. This system enabled non-invasive, real-time monitoring of extracellular pH fluctuations at single-cell level in response to chemotherapeutic treatment (FOLFIRINOX, gemcitabine, paclitaxel). Adopted from Forciniti et al. (2026) under the terms of the Creative Commons Attribution License (CC BY 4.0). **(b)** Dual-dye polystyrene nanosensor combining a pH-inert reference dye (Nile Red) and a pH-responsive fluorescein-based indicator conjugated to a lectin targeting moiety (WGA), enabling ratiometric extracellular pH detection at the cell surface over the range pH 5.5–9.0. Adopted from Kromer et al. (2024) under the terms of the Creative Commons Attribution License (CC BY 4.0). **(c)** Ratiometric fluorescent probe (Cys-Cys-pH) designed for sequential detection of cysteine and intracellular pH changes in pancreatic cancer cells, allowing real-time imaging of intracellular acid-base fluctuations during chemotherapy. Adopted from Qin et al. (2022) under the terms of the Creative Commons Attribution License (CC BY 4.0). **(d)** Silica-based ratiometric optical sensors enabling real-time quantification of endo-lysosomal acidification via confocal microscopy, showing pH-dependent fluorescence shifts from yellow to red upon cellular internalization into acidic intracellular compartments. Adopted from Ouologuem et al. (2025) under the terms of the Creative Commons Attribution License (CC BY 4.0).

### 6.2 Monitoring tumor hypoxia and oxygen gradients

PDAC is characterized by profound dysregulation of oxygen homeostasis, driven by its poorly vascularized and densely fibrotic microenvironment, which restricts perfusion and creates steep metabolic gradients (Kung et al., 2025). Oxygen tension, normally 30–50 mmHg in healthy pancreatic tissue, drops to approximately 2.5 mmHg in tumours, with PDAC being among the most hypoxic cancers, reaching oxygen levels as low as 0.7% and exhibiting highly heterogeneous hypoxic niches (Lohse et al., 2014). These gradients profoundly influence gene expression through hypoxia-inducible factors (HIFs), whose stabilization under low oxygen activates broad transcriptional programs regulating glycolysis, angiogenesis, extracellular matrix remodelling, EMT, immune evasion, and chemoresistance (Arias et al., 2025; Pollard et al., 2014; Nelson et al., 2022; Chen et al., 2023). Because hypoxia is spatiotemporally dynamic and heterogeneous, its accurate monitoring requires advanced sensing platforms capable of mapping intra- and extracellular oxygen at high spatial and temporal resolution. Over the years, various methods have been applied to measure especially extracellular oxygen in biological systems (Dmitriev and Papkovsky, 2012). Traditional direct methods (e.g., electrodes, Seahorse Analyzer) provide bulk measurements but are invasive or costly (Van Der Windt et al., 2016), while indirect methods (e.g., MRI, NADH/NAD^+^ fluorescence imaging) assess oxygen-dependent signals to infer oxygen consumption at the cellular level (Gooz and Maldonado, 2023). More recently, nanotechnology-based optical sensors offer a direct, non-invasive, and highly sensitive way to measure oxygen in real time with micrometer-scale resolution (Yoshihara et al., 2017). These micro- or nano-optical sensors employ fluorescent or phosphorescent oxygen-sensitive probes to provide non-invasive, highly sensitive mapping of oxygen distribution in tissues and cell cultures. Iuele H. and collaborators (Iuele et al., 2024) developed two ratiometric fluorescent microsensors for precise extracellular oxygen monitoring in 3D PDAC models, using low-cost core–shell silica microparticles loaded with oxygen-sensitive dyes (Ru (dpp) or PtOEP) and reference fluorophores (RBITC or A647 NHS-Ester) (Figure 5a). These biocompatible sensors showed rapid, reproducible, and stable responses to oxygen fluctuations and effectively mapped real-time oxygen depletion in 3D alginate hydrogels containing AsPC-1 cancer cells and PSCs, revealing the rapid onset of hypoxia. Parallel advances have enabled detection of intracellular hypoxia markers, such as pimonidazole, HypoxiTRAK™ and HIF-1α staining in spheroids that naturally develop oxygen gradients. Building on this approach, Geyer M. et al. (Geyer et al., 2023) confirmed intracellular hypoxia in a PDAC-on-a-chip model using organoids with or without PSCs by means of a fluorogenic hypoxia probe (Image-iT™ Red), before demonstrating that hypoxic culture conditions (1% O_2_) induce major transcriptional and phenotypic shifts, including KRAS, TGF-β, WNT5a, and EMT pathway activation, and significantly reduce gemcitabine sensitivity while altering responses to MEK, AKT, and EGFR inhibitors (Figure 5b). Altogether, these platforms underscore the need to integrate physiologically relevant oxygen conditions into PDAC research and drug testing (Avelino et al., 2022). Expanding beyond particle-based sensors, electrospun fiber matrices have recently emerged as an alternative scaffold-integrated oxygen sensing platform: ratiometric fluorescent fibers incorporating oxygen-sensitive phosphorescent dyes enable micrometer-spatial-resolution oxygen mapping within advanced cell culture systems, offering compatibility with bioprinted and scaffold-based constructs (Grasso et al., 2024; Grasso et al., 2025a).

**FIGURE 5:**
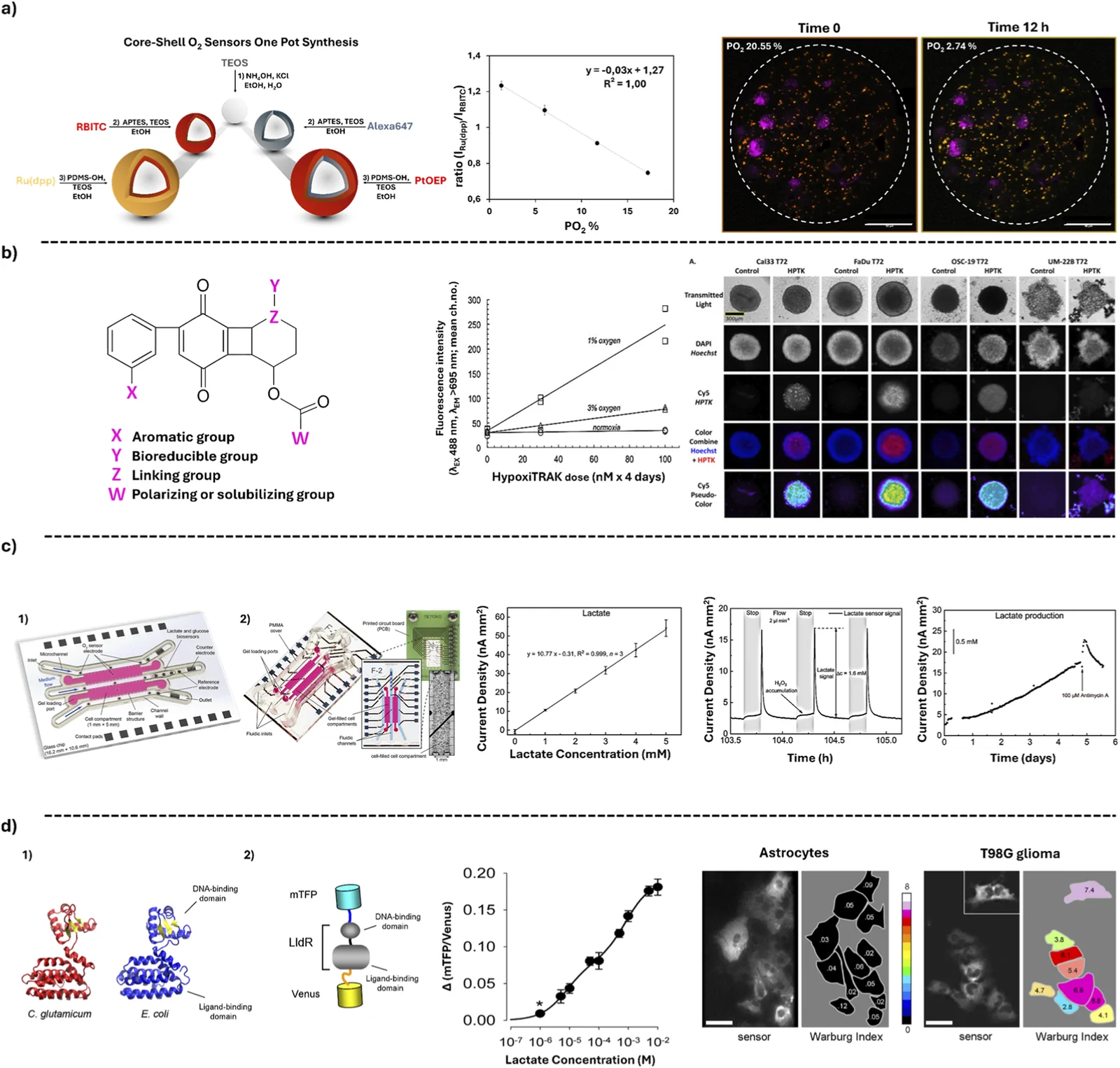
Representative examples of extra- and intracellular oxygen and lactate sensing strategies in cancer models. **(a)** Ratiometric fluorescent microsensors based on core-shell silica microparticles loaded with oxygen-sensitive phosphorescent dyes (Ru (dpp) or PtOEP) and reference fluorophores (RBITC or Alexa647) for real-time extracellular oxygen mapping in 3D alginate PDAC models containing cancer cells (AsPC-1) and pancreatic stellate cells. Reproduced from Iuele et al. (2024) under the terms of the CC BY-NC-ND 4.0 license. **(b)** Microfluidic PDAC-on-a-chip integrating patient-derived organoids cultured with or without pancreatic stellate cells under hypoxic conditions (1% O_2_), demonstrating hypoxia-induced transcriptional reprogramming and reduced gemcitabine sensitivity. Adopted from Geyer et al. (2023) under the terms of the Creative Commons Attribution License (CC BY 4.0). **(c)** Microfluidic organ-on-chip platform with integrated electrochemical biosensor arrays for continuous real-time monitoring of extracellular lactate, glucose, and oxygen in patient-derived tumour spheroids, based on lactate oxidase (LOX)-mediated H_2_O_2_ detection. Adopted from Dornhof et al. (2022) under the terms of the Creative Commons Attribution License (CC BY 4.0). **(d)** Genetically encoded FRET-based lactate sensor (Laconic), built around the bacterial transcription factor LldR sandwiched between fluorescent proteins mTFP and Venus, enabling real-time single-cell quantification of intracellular lactate in the physiologically relevant range (∼1 μM–10 mM). Adopted from San Martín et al. (2013) under the terms of the Creative Commons Attribution License (CC BY 4.0).

### 6.3 Probing lactate dynamics and metabolic gradients

In parallel with hypoxia, impaired perfusion hinders the removal of metabolic by-products, causing lactate accumulation, another defining hallmark of PDAC metabolic rewiring. Excess lactate arises largely from Warburg-driven glycolysis and upregulation of LDHA and the monocarboxylate transporters MCT1 and MCT4 (Heid et al., 2022; Ammar et al., 2023), enabling both lactate efflux from glycolytic clones and uptake by more oxidative tumor or stromal cells, thereby sustaining a metabolic symbiosis (Sandforth et al., 2020). Beyond metabolism, lactate functions as a signalling metabolite: it drives protein lactylation in tumor-associated macrophages, promoting immune suppression and resistance to immune-checkpoint blockade (Sun K. et al., 2025), while fibroblasts use tumour-derived lactate to support extracellular matrix deposition and desmoplasia (Baiskhanova et al., 2025; Yang et al., 2025). Lactate flux also modulates transcriptional and epigenetic programs, including interactions between lactylation and RNA modifications that enhance invasion and metastasis (Liu et al., 2025), as well as MCU-dependent oncogenic signalling (Chen Y. et al., 2025). Because lactate levels are highly dynamic, spatially heterogeneous, and tightly linked to cellular metabolism, accurate assessment requires advanced sensing platforms capable of mapping intra- and extracellular lactate with high spatial and temporal resolution. Over the years, few approaches have been employed to sense lactate production in biological systems and to test lactate-targeting therapies in real-time, using fluorescent or electrochemical biosensors. Dornhof and colleagues (Dornhof et al., 2022) developed a microfluidic organ-on-chip platform integrating electrochemical biosensor arrays to monitor extracellular lactate, glucose, and oxygen in patient-derived tumour spheroids (Figure 5c). The lactate biosensor relies on the LOX-mediated conversion of lactate to H_2_O_2_ within a pHEMA hydrogel immobilized on electrodes, with analyte levels quantified via H_2_O_2_ oxidation currents. The sensor demonstrated high linearity, reversibility, and selectivity within the physiological range, with a detection limit of ∼6.1 µM, enabling long-term, real-time lactate monitoring applicable to various tumour models, including PDAC. To quantify intracellular lactate levels, genetically encoded optical sensors could be employed as ratiometric fluorescent reporters. The Laconic probe is a Förster Resonance Energy Transfer (FRET)–based sensor built around the bacterial transcription factor LldR from the *Escherichia coli*, the teal fluorescent protein mTFP and the green fluorescent protein Venus. It enables real-time, single-cell measurement of intracellular lactate in the physiologically relevant range (from ∼1 μM to 10 mM). Expressed in glioma cancer cell lines (*e.g.*, T98G cells) and normal cells, Laconic has been used in combination with monocarboxylate transporter (MCT) inhibitors to distinguish cells that are net lactate producers from those that consume lactate, thereby quantifying the Warburg effect at the single-cell level (San Martín et al., 2013) (Figure 5d). Moreover, Laconic functionality has been tested in human breast cancer cells (MDA-MB-231 and MCF-7), macrophages, and human embryonic kidney cells (HEK293), proving its reliability in various cell types (Contreras-Baeza et al., 2019; Combs et al., 2024; Jama et al., 2015). Its high specificity, minimal cross-reactivity, and reversible sensing make its a robust tool to map lactate dynamics and metabolic heterogeneity within tumour microenvironments. Beyond genetically encoded sensors, inorganic ratiometric biosensors based on CdTe@CdS quantum dots coupled with lactate oxidase have recently been developed for quantitative fluorescent lactic acid detection, combining high sensitivity with photostability in complex biological matrices (Grasso et al., 2025b). In parallel, pH-sensing nanofibers engineered via electrospinning have enabled single-cell probing of fermentation fluxes and metabolic exchange networks, bridging real-time pH monitoring with the quantification of lactate-coupled proton efflux in cell consortia (Onesto et al., 2023). Collectively, lactate in PDAC represents not merely a metabolic by-product but a dynamic signaling and sensing metabolite that orchestrates tumor-stroma crosstalk, immune suppression, and metabolic plasticity, still requiring deeper investigation. Future integration of biosensing technologies, spatial metabolomics, and single-cell metabolic profiling will be essential to fully delineate lactate’s multifaceted role and to exploit it as a therapeutic vulnerability.

## 7 Comparative considerations and current limitations of metabolic sensing technologies in PDAC models

Although electrochemical, magnetic resonance, and optical sensing approaches have all demonstrated considerable potential for monitoring metabolic dynamics within the microenvironment of PDAC, their suitability strongly depends on the biological question, the complexity of the experimental model, and the balance between spatial resolution, temporal resolution, analytical sensitivity, and invasiveness. Electrochemical sensors provide high analytical sensitivity, rapid response times, and continuous quantitative measurements, making them particularly suitable for bulk extracellular analyses and integration into microfluidic and organ-on-chip platforms. However, they generally require direct contact with the sample, lack spatially resolved information, and may suffer from biofouling, signal drift, and reference electrode instability during prolonged experiments (Ghoneim et al., 2019; Ali, 2017; Steinegger et al., 2020). Magnetic resonance techniques offer the unique advantage of non-invasive volumetric imaging with unlimited tissue penetration and are particularly valuable for *in vivo* studies; nevertheless, their high instrumentation costs and relatively limited spatial resolution reduce their applicability to small-scale in *in vitro* PDAC models (Steinegger et al., 2020; Genicio et al., 2021; Lutz and Bernard, 2019). In contrast, optical sensing technologies have emerged as particularly attractive tools for studying complex three-dimensional PDAC systems because they enable non-invasive, real-time visualization of metabolic gradients with micrometer-scale spatial resolution while remaining compatible with live-cell imaging, spheroids, organoids, hydrogel scaffolds, and organ-on-chip platforms (Hassan Akhtar et al., 2024; Iuele et al., 2025; Yoshihara et al., 2017; Iuele et al., 2024; Grasso et al., 2025b). Their modular architecture further facilitates multiplexed detection of complementary biomarkers, including extracellular pH, oxygen, and lactate, allowing a more comprehensive characterization of tumour metabolic heterogeneity. Similarly, phosphorescence-based oxygen microsensors preserve tissue integrity while providing spatially resolved oxygen maps that cannot be achieved using conventional Clark electrodes or bulk oxygen-consumption assays such as Seahorse analysis (Van Der Windt et al., 2016; Yoshihara et al., 2017; Grasso et al., 2024; Grasso et al., 2025a). For lactate monitoring, electrochemical biosensors currently represent the most mature technology for continuous extracellular measurements owing to their high sensitivity and compatibility with microfluidic platforms, whereas genetically encoded fluorescent probes offer intracellular single-cell resolution but require genetic manipulation, limiting their direct application to patient-derived tumour models (Dornhof et al., 2022; San Martín et al., 2013; Contreras-Baeza et al., 2019; Combs et al., 2024; Jama et al., 2015; Grasso et al., 2025b). Despite these important advantages, several technical challenges remain common to most optical biosensing platforms. First, fluorophore photobleaching during prolonged excitation may progressively decrease signal intensity, although ratiometric sensing strategies substantially mitigate intensity-dependent artefacts and improve measurement robustness (Hassan Akhtar et al., 2024; Grasso et al., 2025b). Accurate quantitative measurements also require careful calibration, as fluorescence and phosphorescence signals may be influenced by temperature, ionic strength, local refractive index, probe microenvironment, and oxygen diffusion kinetics (Wolfbeis, 2015; Wang and Wolfbeis, 2014). In highly desmoplastic PDAC models, the dense extracellular matrix, together with the large dimensions of multicellular spheroids and patient-derived organoids, may further reduce quantitative accuracy because of optical scattering, limited light penetration, and signal attenuation at increasing imaging depths (Lacin and Yildirim, 2024). Furthermore, long-term sensor performance may be affected by gradual probe redistribution and heterogeneous particle distribution, particularly in complex 3D systems (Wolfbeis, 2015). Enzyme-based biosensors may additionally undergo progressive enzyme deactivation, which can compromise signal stability during prolonged measurements (Alam et al., 2018). Biofouling and other matrix-related effects can further affect the analytical performance of lactate biosensors (Alam et al., 2018). Finally, potential intracellular accumulation and the long-term biocompatibility of nanoparticle-based probes should be carefully evaluated when sensors are intended for extended biological experiments (Wolfbeis, 2015). Overall, no single sensing technology can comprehensively address all experimental needs in PDAC research (Table 3). Rather, the optimal sensing strategy should be selected according to the biological question, the complexity of the tumour model, and the required balance between sensitivity, spatial resolution, temporal resolution, imaging depth, and long-term stability. Consequently, future metabolic studies will likely benefit from multimodal sensing platforms integrating complementary pH, oxygen, and lactate biosensors to provide a more complete understanding of PDAC metabolic heterogeneity and its response to therapeutic interventions.

**TABLE 3:** Comparative overview of pH, oxygen, and lactate sensing technologies for PDAC models: advantages, limitations, and applications.

Technology	Advantages	Limitations	Best suited PDAC models	Representative references
Electrochemical pH sensors	High sensitivity, rapid response	Contact-based, drift, biofouling, low spatial resolution	Organ-on-chip	(Ghoneim et al., 2019; Ali, 2017; Steinegger et al., 2020)
Optical pH sensors	High spatial resolution, non-invasive, real-time	Photobleaching, calibration, scattering in thick ECM	Spheroids, organoids, hydrogels	(Hassan Akhtar et al., 2024; Iuele et al., 2025; Grasso et al., 2025b)
Oxygen electrodes	Accurate bulk oxygen consumption	Endpoint/bulk measurements	Cell cultures	(Van Der Windt et al., 2016)
Optical oxygen sensors	Spatial oxygen mapping, live imaging	Photobleaching, diffusion effects, calibration	3D hydrogels, organoids	(Yoshihara et al., 2017; Iuele et al., 2024; Grasso et al., 2024; Wang and Wolfbeis, 2014)
Electrochemical lactate sensors	Continuous monitoring, high sensitivity	Enzyme degradation, biofouling	Organ-on-chip	(Dornhof et al., 2022; Alam et al., 2018)
Genetically encoded lactate sensors	Intracellular, single-cell, reversible	Genetic modification required	Engineered organoids	(San Martín et al., 2013; Contreras-Baeza et al., 2019; Combs et al., 2024; Jama et al., 2015)
Optical lactate probes	Multiplex imaging, non-invasive	Enzyme instability, pH interference	Experimental 3D cultures	(Grasso et al., 2025b; Alam et al., 2018)

## 8 Conclusion

PDAC remains one of the most lethal malignancies, with persistently low survival rates despite decades of research and therapeutic development. Its biological complexity, driven by a dense desmoplastic stroma, extensive cellular heterogeneity, and dynamic metabolic alterations, continues to challenge diagnostic precision and treatment efficacy. As discussed in Section 3, the tight mechanistic interplay between hypoxia, extracellular acidification, and lactate accumulation constitutes a central driver of tumor progression, immune evasion, metabolic adaptation, and chemoresistance in PDAC, underscoring the value of simultaneous multi-parametric sensing of these three metabolic axes (Pan et al., 2025; Sherman and Beatty, 2023; Bogdanov et al., 2022; Cappellesso et al., 2022). Recent advances in experimental modelling and sensing technologies have significantly improved our ability to study PDAC in physiologically relevant contexts. The transition from conventional two-dimensional cultures to sophisticated three-dimensional systems, such as spheroids, organoids, scaffold-based constructs, bioprinted tissues, and microfluidic organ-on-chip platforms, has enabled more accurate reproduction of tumor architecture, mechanical properties, and cell-cell interactions (Narayanan et al., 2021; Cha et al., 2025). In parallel, the integration of ratiometric and optical pH sensors has opened the door to real-time, non-invasive monitoring of the metabolic tumor-stroma interplay (Siciliano et al., 2024). The incorporation of oxygen and lactate sensors alongside pH-sensitive probes now allows for more comprehensive tracking of dynamic metabolic remodeling within PDAC models. Together, these tools are revolutionizing preclinical cancer research by bridging the gap between *in vitro* and *in vivo* observations and providing robust platforms for drug screening, mechanistic studies, and personalized medicine (Godier et al., 2024). Importantly, this review highlights that the greatest potential of these technologies lies not in considering advanced 3D PDAC models and metabolic sensing as independent developments, but in their integration into unified experimental platforms. Combining physiologically relevant 3D models with dynamic metabolic monitoring enables longitudinal and functional characterization of the TME, providing a more comprehensive understanding of PDAC biology than conventional endpoint analyses alone. By integrating advanced biosensing technologies with 3D PDAC models, metabolic sensing has the potential to bridge the gap between experimental cancer models and clinically relevant precision oncology, supporting improved drug screening, patient stratification, and the development of personalized therapeutic strategies.

## 9 Future perspectives

Despite the remarkable progress achieved in recent years, several challenges still need to be addressed before metabolic sensing technologies can be fully translated into clinically relevant platforms for PDAC research. Although advanced *in vitro* 3D models have significantly improved the recapitulation of tumor architecture and cell–cell interactions, reproducing the full biochemical complexity of the PDAC tumor microenvironment, including spatial and temporal gradients of oxygen, nutrients, lactate, pH, cytokines, growth factors, and mechanical cues, remains a major challenge. Moreover, the lack of standardized protocols for model generation, sensor integration, data acquisition, and data analysis continues to limit reproducibility and cross-platform comparison, representing one of the principal barriers to clinical translation. Overcoming these limitations will require multidisciplinary strategies that integrate functional metabolic sensing with complementary molecular, imaging, and computational technologies, ultimately bridging the gap between advanced preclinical models and clinically actionable precision medicine (Dahl-Jensen et al., 2018). In this context, the integration of metabolic sensing with spatial transcriptomics, spatial proteomics, single-cell RNA sequencing (scRNA-seq), and metabolomics is expected to provide a more comprehensive understanding of PDAC heterogeneity. While optical and electrochemical biosensors enable continuous, real-time monitoring of extracellular pH, oxygen availability, and lactate dynamics, these functional measurements alone cannot fully explain the molecular mechanisms driving metabolic adaptation. Combining biosensor-derived metabolic maps with spatial transcriptomics and proteomics analysis would allow localized metabolic alterations to be directly correlated with gene-expression programs, protein signatures, stromal activation, and immune-cell organization within the tumor microenvironment (Longo et al., 2021; Marx, 2021). Likewise, scRNA-seq can identify transcriptionally distinct tumor, stromal, and immune-cell populations associated with specific metabolic states, thereby linking functional biosensor measurements with cellular heterogeneity, whereas metabolomics provides complementary biochemical validation by characterizing metabolic pathway activity and metabolite abundance (Armingol et al., 2021). Rather than representing independent technologies, these approaches should be viewed as highly complementary, collectively enabling the generation of a multidimensional spatiotemporal atlas of PDAC metabolism that integrates functional, molecular, and cellular information. Such an integrated framework could facilitate the identification of patient-specific metabolic vulnerabilities, improve biomarker discovery, and support more accurate prediction of therapeutic response. Beyond molecular integration, artificial intelligence (AI) and advanced computational modelling are expected to further enhance the analytical and predictive capabilities of metabolic sensing technologies. Experimental studies of 3D PDAC models integrated with optical biosensors generate highly complex datasets comprising time-resolved fluorescence signals, spatial metabolic gradients, high-content microscopy images, and longitudinal treatment responses that cannot be fully exploited using conventional analytical methods. Machine learning and deep learning algorithms are increasingly being employed to automate image segmentation, quantify spatial heterogeneity, extract high-dimensional imaging features, and identify complex relationships between metabolic alterations and therapeutic response (Perez-Lopez et al., 2024). Furthermore, integrating biosensor-derived measurements with quantitative image analysis, clinical information, and multi-omics datasets could enable predictive computational models to identify metabolic signatures associated with drug sensitivity or resistance before measurable phenotypic changes occur, thereby improving patient stratification and facilitating high-throughput drug screening. Looking further ahead, AI-assisted analysis may support the development of digital biomarkers and patient-specific digital twins, in which patient-derived 3D models, real-time metabolic sensing, and multimodal molecular profiling are integrated to simulate therapeutic responses and optimize treatment selection prior to clinical intervention (Wu et al., 2023). Ultimately, the convergence of patient-derived 3D PDAC models, real-time metabolic sensing, multi-omics technologies, and AI-driven computational analysis has the potential to transform metabolic biosensing from a descriptive experimental approach into a truly predictive platform for precision oncology, bridging the gap between experimental models and clinical decision-making and enabling the identification of actionable metabolic vulnerabilities and personalized therapeutic strategies for patients with pancreatic cancer.
